# Genetics and Pathogenesis of Feline Infectious Peritonitis Virus

**DOI:** 10.3201/eid1509.081573

**Published:** 2009-09

**Authors:** Meredith A. Brown, Jennifer L. Troyer, Jill Pecon-Slattery, Melody E. Roelke, Stephen J. O’Brien

**Affiliations:** National Cancer Institute, Frederick, Maryland, USA (M.A. Brown, J. Pecon-Slattery, S.J. O’Brien); SAIC-Frederick, Inc., Frederick (J.L. Troyer, M.E. Roelke)

**Keywords:** Coronavirus, infectious peritonitis, feline, virulence, genetic, marker, viruses, Maryland, USA, research

## Abstract

Feline coronavirus (FCoV) is endemic in feral cat populations and cat colonies, frequently preceding outbreaks of fatal feline infectious peritonitis (FIP). FCoV exhibits 2 biotypes: the pathogenic disease and a benign infection with feline enteric coronavirus (FECV). Uncertainty remains regarding whether genetically distinctive avirulent and virulent forms coexist or whether an avirulent form mutates in vivo*,* causing FIP. To resolve these alternative hypotheses, we isolated viral sequences from FCoV-infected clinically healthy and sick cats (8 FIP cases and 48 FECV-asymptomatic animals); 735 sequences from 4 gene segments were generated and subjected to phylogenetic analyses. Viral sequences from healthy cats were distinct from sick cats on the basis of genetic distances observed in the membrane and nonstructural protein *7b* genes. These data demonstrate distinctive circulating virulent and avirulent strains in natural populations. In addition, 5 membrane protein amino acid residues with functional potential differentiated healthy cats from cats with FIP. These findings may have potential as diagnostic markers for virulent FIP-associated FCoV.

## Abstract 2

Coronavirus sequence analyses demonstrate distinctive circulating strains in natural populations.

Feline infectious peritonitis (FIP) is an uncommon, fatal, progressive, and immune-augmented disease of cats caused by feline coronavirus (FCoV) infection. Although FCoV is common in most domestic, feral, and nondomestic cat populations worldwide (seroprevalence 20%–100%), FIP will develop in <10% of FCoV seropositive cats (*1*–*4*). FIP tends to occur most frequently in cats <2 years of age or, less commonly, in geriatric cats (*4*,*5*). The clinical manifestations of FCoV infection can be either a pathogenic disease, FIP (cases infected with feline infectious peritonitis virus [FIPV]) and, more commonly, a benign, or mild enteric infection (feline enteric coronavirus [FECV] asymptomatic) (*6*,*7*). Specific genetic determinants of these clinical outcomes have yet to be discovered. There is no effective treatment, vaccine, or diagnostic protocol that can discriminate the avirulent FECV from FIPV.

FIP pathology is characterized typically by severe systemic inflammatory damage of serosal membranes and widespread pyogranulomatous lesions, which occurs in the lungs, liver, lymph tissue, and brain (*8*). Evidence suggests that the host immune system is crucial in this pathogenesis; profound T-cell depletion from the periphery and lymphatic tissues and changes in cytokine expression are observed in end-stage FIP (*9*,*10*). The clinical finding of hypergammaglobulinemia-associated FIP is indicative of virus-induced immune dysregulation (*11*).

Viral genetic determinants specifically associated with FIPV pathogenesis have yet to be discovered. An in vivo mutation transition hypothesis postulated that de novo virus mutation occurs in vivo, giving rise to virulence (*12*,*13*). The precise nature of the mutation responsible for pathogenesis has not been identified, although studies have suggested sequence differences in the spike protein (*14*), nonstructural protein (NSP) 7b, and NSP3c (*13*) as disease determinants. Together with in vitro studies describing the FIPV strains affinity for macrophages in contrast to FECV strains (*15*), the hypothesis was extended to propose that the enteric coronavirus (FECV) undergoes a mutational shift in the gastrointestinal system, thus allowing infection of macrophages, systemic dissemination, and fatal disease manifestation (*12*,*13*). However, attempts to use engineered chimeric viruses designed to identify the operative virulence determinants have been unsuccessful (*16*). Furthermore, circulating FCoVs found in different tissues of FCoV-infected asymptomatic cats were indistinguishable (*17*,*18*).

The in vivo mutation hypothesis of FIPV pathogenesis is widely cited, although it has never been explicitly confirmed. Mutational transition of HIV-1 has been demonstrated in AIDS patients, in which mutation of envelope residues alters coreceptor use from CCR5 to CXCR4, a prelude to the collapse of the CD4-bearing lymphocyte population (*19*). Similarly, key amino acid changes in the porcine coronavirus spike gene lead to increased virulence in the coronavirus transmissible gastroenteritis virus, a fatal disease causing high rates of illness and death in young pigs (*20*–*22*).

An alternative circulating virulent-avirulent FCoV hypothesis of viral pathogenesis suggests that distinctive benign and pathogenic strains of FECV circulate in a population, and that the disease will develop only in those persons infected by the virulent strains. Dengue virus may offer an example because it has been shown that 4 distinctive viral strains circulate worldwide, and severe hemorrhagic fever develops in persons exposed sequentially to distinct strains (*23*). Zoonotic equine Venezuelan encephalitis virus also displays circulating virulent and avirulent strains, which through interaction with ecologic and epidemiologic factors, contribute to or constrain the disease incidence (*24*).

This study aimed to systematically test evolutionary predictions of the in vivo mutation hypothesis versus the circulating virulent/avirulent hypothesis in the pathogenicity of FIP in the cat. We developed a study of naturally occurring FECV and FIPV using molecular genetic tools by collecting samples from field cases of FIP (cases) and FECV-positive but asymptomatic cats (controls). Cases were infected with feline coronavirus (FCoV) and had the clinical disease of feline infectious peritonitis (FIP). Controls were also infected with FCoV, but were clinically asymptomatic (FECV-asymptomatic). The prediction was that phylogenetic analysis of viral gene sequences would demonstrate paraphyly for FIP case-cats and FECV-asymptomatic cats if the in vivo mutation hypothesis was supported, and monophyly of the 2 if the circulating virulent/avirulent hypothesis was supported (Figure 1). Additionally, we surveyed the viral genetic diversity and dynamics and determined genetic signatures associated with pathogenesis in FIP.

**Figure 1:**
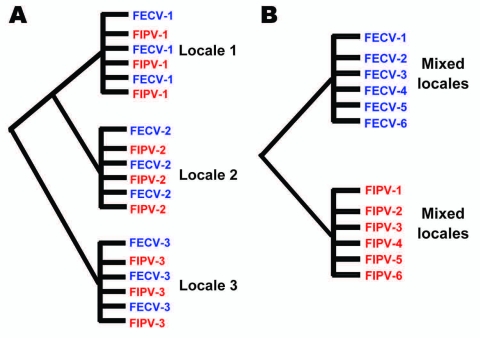
Alternative phylogenetic predictions of the in vivo mutation hypothesis versus the dual circulating virulent/avirulent hypothesis. A) The in vivo mutation transition hypothesis predicts paraphyly of feline infectious peritonitis (FIP) cases and feline enteric coronavirus (FECV) asymptomatic feline coronavirus (FCoV) isolates). B) The circulating virulent/avirulent strain hypothesis predicts reciprocal monophyly of FIV-cases versus FECV asymptomatic. Numbers represent individual cat (or locale), which is either FIPV case (red) or FECV asymptomatic (blue). Evidence presented in this article supports the circulating dual virulent and avirulent strains.

## Materials and Methods

### Sampling

A total of 56 live, euthanized, or recently deceased domestic cats were clinically examined and sampled in Maryland veterinary hospitals during 2004–2006 (Appendix Table 1). Blood (3–6 mL) was collected routinely by venipuncture from manually restrained or anesthetized domestic cats. Feces were obtained from the rectum by cotton swab and frozen in 0.5 mL of phosphate-buffered saline. Cats from 1 (Weller Farm) of 6 farms were micro-chipped (AVID, Folsom, LA, USA) for identification for repeat sampling of individual cats. Samples were collected in full compliance with specific federal permits (Convention on International Trade in Endangered Species; Endangered and Threatened Species) issued to the National Cancer Institute by the US Fish and Wildlife Service of the Department of the Interior.

For euthanized and recently deceased cats, gross necropsy examination and sample collection were performed within 2 hours of death. Samples from liver, spleen, mesenteric lymph node, kidney, jejunum, and colon were taken, fixed in 10% buffered formalin, and routinely embedded in paraffin. Sections (5 μm) were stained with hematoxylin and eosin (HE). Tissues were also flash frozen in liquid nitrogen (–220°C) for RNA extraction and stored at either –220°C or –70C°.

### Clinical Hematologic and Biochemical Analysis

For complete blood counts, fresh (<12 hours) whole-blood samples were assessed by Antech veterinary diagnostic laboratory by using an automated cell counter (Avid Cell-Dyn 3500; Abbott Laboratories, Abbott Park, IL, USA). Biochemical analysis (Hitachi 717 Clinical Chemistry Analyzer; Roche Diagnostics, Indianapolis, IN, USA) and ELISA for feline immunodeficiency virus (FIV; Petchek FIV ELISA, Idexx Laboratories, Westbrook, ME, USA), and coronavirus (Virachek CV, Synbiotics Corp., San Diego, CA, USA) antibodies were also performed.

### Pathologic and Immunohistochemical Analysis

**Figure 2:**
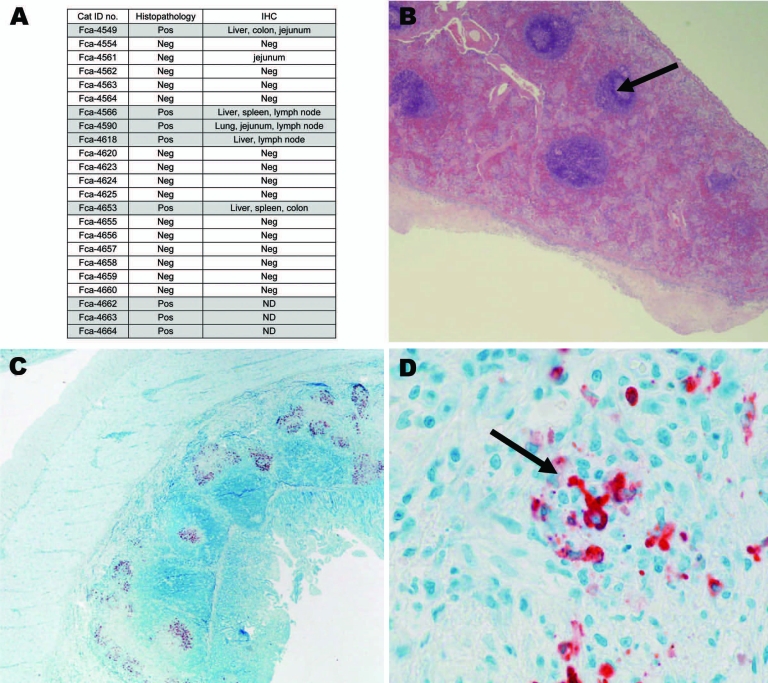
A) Histopathologic and immunohistochemical (IHC) results from 23 necropsied cats positive for antibodies against feline coronavirus. Liver, lung, spleen, colon, jejunum, stomach, heart, kidney, lymph node were evaluated by IHC. Feline infectious peritonitis (FIP) cases are highlighted in gray. Pos, positive; Neg, negative; ND, not done. B) Representative tissues from cat no. FCA-4653, spleen (histopathologic) showing granuloma (arrow); magnification ×20. C) Representative tissues from cat no. FCA-4590, small intestine (IHC); magnification ×20. D) Red staining indicates binding of coronavirus antibody (CoV p56, arrow); magnification ×100.

HE-stained slides of spleen, liver, lymph node, intestine, and kidney sections were evaluated for evidence of granulomatous and pyogranulomatous lesions (National Cancer Institute Laboratory Animal Sciences Program, Frederick, MD, USA). Formalin-fixed sections (3 μm thick) were cut from paraffin blocks and placed on glass slides for immunohistochemical (IHC) testing, as previously described, with CoV p56, a cross-reacting antibody for the demonstration of feline coronavirus (FECV and FIPV biotypes) (*9*,*10*) (Washington Animal Disease Diagnostic Laboratory, Pullman, WA, USA) (Figure 2).

### RNA Extraction and Reverse Transcription

RNA from 160 μL ascites fluid or frozen feces suspended 10% in phosphate-buffered saline was extracted by using the QIAamp virus RNA mini kit (QIAGEN, Valencia, CA, USA) following the manufacturer’s instructions. RNA from tissue was extracted from ≈60 mg of frozen liver, lung, spleen, colon, jejunum, or lymph node by using RNAeasy (QIAGEN) following the manufacturer’s instructions. Extracted RNA was eluted in 35 μL of RNase-free water and stored at –70ºC. cDNA was reverse transcribed using 9 μL of eluted RNA (10 pg–5 μg) in an initial 12-μL reaction mixture containing 50 ng of random hexamers and 0.5 mmol/L of dNTPs. After incubation at 65ºC for 5 min to denature the RNA, 10 mmol/L of dithiothreitol, 5× Synthesis Buffer, 40 U of RNaseOUT, and 15 units of Thermoscript RT were added on ice (Invitrogen, Carlsbad, CA, USA). Reaction mixtures were incubated in thermocycler at 25ºC for 10 min, followed by 50ºC for 30 min. cDNA was stored at –20ºC.

### PCR

**Figure 3:**
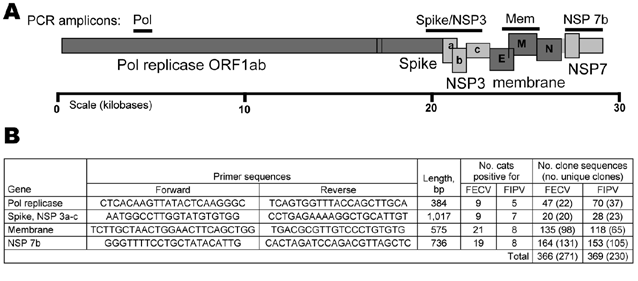
A) Feline coronavirus genome indicating PCR products obtained (bars). Structural proteins are shaded in dark gray; nonstructural proteins are shaded in light gray. B) Forward and reverse primers used to amplify virus segments are listed in 5′ → 3′ orientation. The number of source cats and cloned sequences generated from feline infectious peritonitis (FIP) cases and feline enteric coronavirus (FECV) asymptomatic cats are presented. Pol, polymerase; NSP, nonstructural protein; FIPV, feline infectious peritonitis virus.

Primers amplifying 7b (736 bp), membrane protein (575 bp), polymerase (386 bp), and spike-NSP3 (1,017 bp) (Figure 3) were designed based on available feline coronavirus sequence (*1*,*12*,*13*). PCR was performed by using 2 μL of cDNA in a 50-μL reaction containing 50 mmol/L KCl, 10 mmol/L Tris-HCl (pH 8.3), 1.5 mmol/L MgCl_2_, 0.25 mmol/L concentrations of dNTPs (dATP, dCTP, dGTP, and dTTP), 2 mmol/L concentrations of each primer, and 2.5 units of Platinum Taq DNA polymerase (Invitrogen). PCR was conducted on a geneAmp PCR system 9700 thermocycler (Applied Biosystems, Foster City, CA, USA) with the following touchdown conditions: 2 min at 94ºC then a touchdown, always starting with 20 sec at 94ºC, then 30 sec at 60ºC (3 cycles), 58ºC (5 cycles), 56ºC (5 cycles), 54ºC (5 cycles), 52ºC (22 cycles), and then 1 min at 72ºC for extension, and with a final extension at 72ºC for 7 min and a hold at 4ºC. PCR products were visualized by electrophoresis on a 1% agarose gel and primers and unincorporated dNTPs were removed by using Microcon YM (Millipore, Billerica, MA, USA).

### Cloning and Sequencing

Representative PCR products were cloned and sequenced (Figure 3, panel B). Cloning was performed by using a TOPO-TA cloning kit (Invitrogen) according to the manufacturer’s instructions. Plasmid DNA was isolated from 1–47 clones from each reaction product (Agencourt CosMCPrep; Agencourt Bioscience Corporation, Beverly, MA, USA). The presence of the correct sized insert was verified by restriction enzyme digestion (*Eco*RI), and sequences were obtained from clones with the correct insert by using standard ABI BigDye terminator reactions (Applied Biosystems). Anticontamination measures were taken at all steps of reverse transcription–PCR (RT-PCR) amplification and post-PCR processing.

### Phylogenetic Analysis

Sequences from *pol 1a, spike-NSP3, membrane,* and *NSP7b* were analyzed separately. Nucleotide sequences were compiled and aligned for subsequent phylogenetic analysis by using ClustalX (*25*) and verified visually (*26*). Analyses involved producing a phylogenetic tree of viral gene sequences based upon the following approaches: minimum evolution, maximum parsimony, and maximum likelihood in PAUP (*27*). Modeltest was used to estimate the optimal model of sequence evolution, and these settings were incorporated into subsequent analyses (*28*). Minimum evolution trees were constructed from models of substitution specified by Modeltest, with starting trees obtained by neighbor joining followed by application of a tree-bisection-reconnection (TBR) branch-swapping algorithm during a heuristic search for the optimal tree. Maximum parsimony analysis employed a heuristic search of starting trees obtained by stepwise addition followed by TBR. Maximum-likelihood parameters specified by Modeltest selected the general time-reversible model of substitution, included empirical base frequencies, and estimated rate matrix and corrected for among-site rate variation (gamma distribution). A bootstrap analysis using 1,000 iterations was performed for maximum parsimony and minimum evolution and 100 iterations by using the nearest neighbor interchange branch-swapping algorithm for maximum likelihood. Amino acid residue alignments were generated using MacClade 3.05 (*26*) and ClustalX (www.softpedia.com/get/Science-CAD/Clustal-X.shtml).

Variable sites and parsimoniously informative sites were computed in MEGA version 3.0 (*29*). Pairwise comparisons of genetic distances were performed in PAUP and the mean and range of genetic distances were calculated in Excel (Microsoft, Redmond, WA, USA). The sequences of FCoV *pol 1a, membrane, NSP 7b,* and *spike-NSP3* were deposited in GenBank under accession nos. EU663755–EU664317.

## Results

During 2004–2006, fifty-six domestic cats with suspected FIP or exposure to infected FIP cats from Maryland farms and veterinary hospitals were sampled (Appendix Table 1). All samples producing RT-PCR products were from cats positive for antibodies against FCoV (titers >25). Thirty-six sampled cats were from the Weller farm where several individual cats were sampled once per year for the 3-year study period. Healthy and recently deceased or euthanized cats were included from the Ambrose farm (n = 7), Palmer Veterinary Hospital (n = 3), Frederick County Animal Shelter (n = 7), Seymour farm (n = 1), and the New Market Animal Hospital (n = 2). Fca-4590 from the Weller farm is an important FIP case because samples were obtained on May 20, 2004, when the cat was clinically healthy (predisease) and again on December 22, 2004, when FIP developed in the cat and it died (postdisease).

Necropsies were performed on 23 cats that died or were euthanized due to suspected FIP. Most of the necropsied cats were FCoV antibody positive (Appendix Table 1). Eight cats were classified as FIP cases based on histopatholgic findings (Fca-4549, Fca-4566, Fca-4590, Fca-4618, Fca-4653, Fca-4662, Fca-4663, and Fca-4664) (Figure 2; Appendix Table 1). The presence of pyogranulomatous lesions at histology evaluation was sufficient for designation of an FIP case. Additionally, 5 of the 8 FIP cases were evaluated by IHC testing. Multiple tissues were positive by IHC in each of these cats. One cat (Fca-4561) was IHC positive only in the jejunum and negative by histopathologic analysis on all tissues, therefore it was classified as FECV asymptomatic. The FCoV-seropositive necropsied cats with no characteristic FIP histopathologic changes and IHC lesions were classified as FECV asymptomatic (Appendix Table 1; Figure 2). Healthy cats were classified as FECV asymptomatic if they had normal results on physical examinations, were FCoV antibody positive (titer >25) but not lymphopenic (<1.5 cells/μL), or were monitored until 2007 and known to be free of FIP disease (Appendix Table 1).

RT-PCR was attempted with 4 primer pairs designed from FCoV genes for all cats (Figure 3, panel B). Of the 82 samplings from 56 cats, 42 samplings amplified virus with at least 1 primer pair yielding a 51% rate of recovery of viral sequence (Appendix Table 1). From 8 cats with clinical FIP and 23 FECV-asymptomatic cats, amplification from the 4 viral regions produced 735 cloned viral gene segments that resulted in 501 unique gene sequences ( Appendix Table 2; Figure 3, panel B).

**Figure 4:**
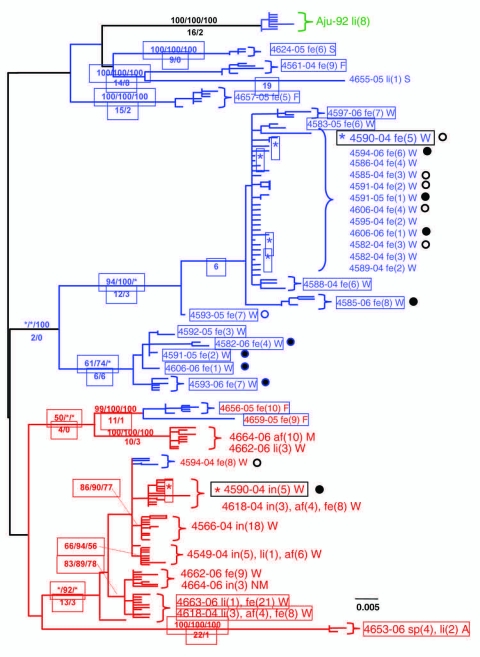
Maximum-likelihood (ML) phylogenetic tree of unique sequences from 3 feline coronavirus (FCoV) genes membrane, nonstructural protein 7b (*NSP 7b*), and *spike-NPS3* (see Figure 3) gene sequences showing monophyly correlating to disease status. Cloned sequences from feline infectious peritonitis (FIP) cases are shown in red; feline enteric coronavirus (FECV) asymptomatic cats are shown in blue, and FCoV virulent strain from Aju-92 (cheetah) is in green. Number of cats and number of clones assessed are listed in Figure 3, panel B. A) *membrane* 575-bp sequences (ML –ln L = 3086.20787 best tree found by maximum parsimony [MP]: length = 493, confidence interval [CI] = 0.551724, retention index [RI] = 0.0926505); B) *NSP 7b* 736-bp sequences (ML –ln L = 4556.60497 best tree found by MP: length = 452, CI = 0.608, RI = 0.942; C) *spike-NSP3* 1017-bp sequences (ML –ln L = 2804.53198 best tree found by MP: length = 280, CI = 0.800, RI = 0.954). The number of FIP cases and FECV asymptomatic cats and number of cloned sequences is indicated in parenthesis in the key. Each sequence is labeled as follows: first letter indicates source farm (W, Weller Farm; F, Frederick Animal Shelter; S, Seymour Farm; M, Mount Airy Shelter; A, Ambrose Farm), 4-digit cat identification number, tissue source (fe, feces; af, ascites fluid; co, colon; li, liver; sp, spleen; in, intestine; je, jejunum; ln, lymph node), 2-digit year (e.g., 04 = 2004), and the number of clones for each sequence. Bootstrap values are shown (maximum parsimony/minimum evolution/maximum likelihood) above branches. Where maximum likelihood tree was congruent with maximum parsimony tree, branch lengths are indicated below branches; the number of homoplasies is in parenthesis after the branch length. Virus sequence obtained from cat no. 4590 in May 2004 and at the time of death due to FIP in December 2004 is indicated by box. Scale bars indicate substitutions/site.

Phylogenetic analysis of the cloned virus sequences from 3 Maryland locales sampled during 2004–2006 showed specific patterns of viral dynamics. First, gene sequences from healthy cats infected with FECV displayed a monophyletic cluster pattern that was generally distinctive from cats diagnosed with FIP in the *membrane*, *NSP 7b,* and *spike-NSP3* gene segments (Figure 4). For example, every FCoV gene sequence for the membrane gene from FIP cases fell within a major cluster consisting of 3 principal clades (Figure 4). By contrast, 127/154 (82%) virus gene sequences from FECV-asymptomatic cats sorted in 2 separate clades that were distinct (100 bootstrap statistical support) from the viral gene sequences of FIP cases (Figure 4). Similar reciprocal monophyly of 140 *NSP7b* sequences was obtained for FIP cases versus FECV-asymptomatic cats (Figure 4). A consistent disease driven phylogeographic sorting was also observed for the 1,017-bp sequence spanning the *spike-NSP3* genes, albeit with less statistical resolution, likely because of evolutionary constraints on gene divergence in this region (Figure 4). Together the remarkable reciprocal monophyly in these 3 genes supports the predictions of the circulating virulent-avirulent strain hypothesis illustrated in Figure 1.

Samples from 1 cat, Fca-4590, were particularly informative. The virus was isolated from the cat predisease, and then again 7 months later postdisease. Fca-4590 was asymptomatic but infected with FECV in May 2004. FCoV sampling from that month showed strong (high bootstrap) affiliation with the FECV-asymptomatic clades for the membrane and the *NSP7b* genes. However, virus isolated 7 months later in December 2004 after FIP developed in Fca-4590 fell within the FIP-case clades (also with high bootstrap), and was indistinguishable from FCoV isolated from other cats with FIP. This finding suggested that the pathogenic FIP-case type of FCoV infected this cat subsequent to its infection with an avirulent FECV and apparently replaced it.

Tissue-specific differentiation within each cat was minimal (Figure 4). By contrast, there were notable locale-specific distinctions within the sick and healthy cats (Figure 4). For example, the FECV strains in asymptomatic cats from the Weller household were associated together in a major FECV subclade; strains in cats from the Frederick Animal Shelter were classified in a different subclade, nested within the FECV-asymptomatic clade (Figure 4). The archival FCoV virulent strain (Aju-92), isolated from cheetahs in Oregon in 1982 (*30*), defined a phylogenetic lineage distinctive from the FIP and FECV-asymptomatic clades resolved in the Maryland domestic cats (Figure 4).

Nucleotide sequences of *membrane* and *NSP 7b* generated in this study were translated to amino acid sequences (Appendix Figure 1). Relative to pathogenesis, 5 informative amino acid sites were found in the membrane protein at positions 108, 120, 138, 163, and 199 (based on reference sequence for TGEV GenBank no. NP058427) (*22*), giving rise to 6 composite genotypes potentially diagnostic of FIP cases versus FECV-asymptomatic cats ( Appendix Table 2). Among the 8 cats with FIP, 19 FECV-asymptomatic cats, and 1 cheetah with FIP, 6 composite genotypes were identified based on these 5 diagnostic sites ( Appendix Table 2).

All domestic cats with FIP diagnosed by pathologic or immunohistochemical changes displayed the amino acid signature of either YIVAL (I) or YIIAL (II); infected cats without clinical FIP had the HIIVI (III), HIIVL (IV), HVIAL (V), YVVAL (VI), or YIVAL (I) haplotype. No FIP cases had haplotype III, IV, V, or VI, whereas 3 FECV-asymptomatic cats carried the YIVAL signature found predominately in FIP cases (Fca-4594, 4624, and 4657;  Appendix Table 2). Of these, 2 cats (4624 and 4657) were euthanized at the time of sampling (all euthanized FECV-asymptomatic cats are highlighted in light green in  Appendix Table 2); therefore, whether clinical FIP would have later developed in these cats is unknown. The other exception, cat 4594, was sampled twice (in 2004 and again in 2006); the switch in genetic signature from YIVAL in 2004 to HIIVI in 2006 may indicate that this cat was able to clear a virulent FIPV strain after the 2004 sampling and become reinfected with an avirulent strain by 2006. Although a strong phylogenetic signal differentiating FIP cases from FECV-asymptomatic cats was seen in *NSP 7b* (Figure 4), no diagnostic amino acid changes specific to FIP cases vs. FECV-asymptomatic controls were found in the *NSP 7b* nucleotide or amino acid alignments. In contrast to the monophyletic findings in the *membrane,* *NSP 7b,* and *spike*-*NSP3* genes, cloned viral sequences of *pol 1a,* were paraphyletic in terms of disease phenotype (Appendix Figure 2).

## Discussion

Infection with FCoV is common in cats throughout the world, although in most cats the virus causes no clinical signs. However, in some cats, FCoV infection is associated with the development of the progressive and fatal disease manifestation of FIP. This disease is among the most serious viral infections in cats, not only because of its fatal nature, but also because of the difficulties in diagnosing FIP antemortem and controlling the spread of FCoV. We have presented a molecular virologic study of naturally occurring FCoV infection and phylogenetic analysis of the cloned virus sequences obtained from the *membrane,* *NSP 7b, spike-NSP3,* and *pol 1a* genes isolated from domestic cats located in Maryland households infected with FCoV during 2004–2006. We observed predominately monophyletic clustering of strains correlating with disease phenotype in *membrane and NSP 7b* genes consistent with the circulating virulent/avirulent strain hypothesis of FIP pathogenesis, which calls into question the in vivo mutation hypothesis.

The amino acid alignments presented in Appendix Figure 1 clearly demonstrate that in the FIPV cases included in this study the genotypes correlated with disease phenotype are ancestrally derived and not the result of a few de novo mutations. Given the clear genetic differentiation between viruses from FIP cases and FECV asymptomatic cats in multiple gene segments, we infer that cats become reinfected with new strains of FCoV from external sources, rather than by in vivo mutations. Cats in our study were not co-infected with multiple strains of FECV and FIPV at the same time and were generally infected with one predominant virus strain. Two exceptions to this finding in our study were cats with cases of FIPV (Fca-4662 and 4664) that from which distinct gastrointestinal (feces or intestine) and systemic (liver and/or ascites fluid) viral isolates were obtained, which indicates that in vivo superinfection does occur (Figure 4;  Appendix Table 2).

**Figure 5:**
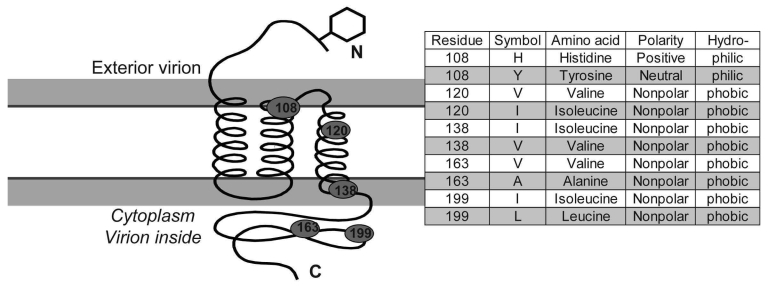
Diagram of membrane protein containing 3 transmembrane helices, an external N terminus and an internal carboxy terminus. Approximate position of 5 variable diagnostic amino acid sites (see Table 2) as determined by sequence comparison to severe acute respiratory syndrome coronavirus (*32*). Amino acid residue, polarity, and hydrophobicity or hydropholicity is stated.

A role of the membrane protein in FIP pathogenesis seems likely, given its known functions in other coronaviruses. The membrane protein is the most abundant structural protein with important functions in virus budding and with cell-mediated host immunity (*31*). The specific functions of the membrane protein amino acid sequences have been determined in severe acute respiratory syndrome (SARS)–CoV (*32*). Aligning the sequences from this study with SARS-CoV, the first diagnostic amino acid site 108 aligns to a site just upstream from the second transmembrane helix (Appendix Figure 1). A tyrosine at position 108, which is found in all FIPV biotypes and shared among SARS-CoV, has a neutral polarity (in contrast to a histidine there, found in most FECV biotypes, which have a positive polarity) and may play a role in the stability of the virus within the membrane. Site 120 aligns within the third transmembrane helix, site 138 aligns just downstream to the transmembrane helice, site 163 aligns within the C-terminus, which projects inside the virus particle, and site 199, also within the C-terminus domain, aligns within a defined SARS-immunodominant epitope (*32*) (Figure 5).

The demonstration of 6 naturally occurring composite genotypes based on 5 variable sites in the membrane protein amino acid alignment that are highly correlative with disease phenotype ( Appendix Table 2) offers specific opportunities for developing diagnostics and for the preventive management of this disease. By extending this study to additional cat populations in disparate geographic locations, designing chimeric FCoV challenge experiments, and investigating host genetic correlations with pathogenesis, we will be able to further discern the causative factors in FIP pathogenesis. Fca-4594, which was infected with the disease-associated genotype composite without succumbing to FIP, suggests additional requirements for viral pathogenesis. As has been suggested in the outbreak of FIP in a colony of captive cheetahs (*33*), host immune genetics may play a role.

Both the viral strain and host immune genes contribute to disease progression and virus-related death, such as AIDS progression in HIV infection. With the recent publication of the full cat genome sequence (*34*) and the viral genotype composites described here, new genomic tools are now available to proceed with both viral and host genetic association studies in the pathogenesis in FCoV infection, a model for coronavirus infection in humans, such as SARS-CoV.

## Supplementary Material

Appendix Table 1 Clinical, demographic, and FCoV viral RT-PCR success data from 56 domestic cats sampled in Maryland, USA, 2004-2006*

Appendix Table 2 Genotype composites of 8 FIPV and 19 FECV domestic cats in Maryland, USA, sampled 25 times at 5 variable amino acids in the FCoV membrane protein*

Appendix Table 3 Additional clinical data from 56 domestic cats sampled in Maryland, USA, 2004-2006*

Appendix Figure 1 Alignment of variable sites of unique amino acid sequences of membrane and nonstructural protein 7b (NSP 7b) genes from feline infectious peritonitis virus (FIPV) cases (gray shaded), from feline enteric coronavirus (FECV) asymptomatic cats, from feline coronavirus (FCoV)-Aju, and reference sequences for severe acute respiratory syndrome coronavirus (CoV), MHV-1, IBV-Beu, BVC-K, HcoV-229E, TGEV-Purdue, and FCoV 79-1146 (GenBank accession nos. P59596, AB587268, P69602, BAF75636, P15422, PO4135, and P25878, respectively). FCoV reference sequences for FECVUCD, FIPVUCD1, FIPV791146, and FIPVUCD3 are also included. Diagnostic sites are highlighted in the membrane. For membrane, cat ID and 2-digit year of sampling is listed and the number of original clones is in parenthesis; the frequency of unique amino acid sequences is reported in column 2. No diagnostic sites were found correlating with FIPV and FECV biotype in NSP 7b.

Appendix Figure 2 Midpoint rooted maximum likelihood (ML) tree of unique pol1a 386-bp sequences (ML -ln L = 1300.12586 best tree found by maximum parsimony: length = 125, confidence interval = 0.832, retention index = 0.926). Cloned sequences from feline infectious peritonitis (FIP) cases are shown in red, feline enteric coronavirus (FECV) asymptomatic cats are shown in blue, and feline coronavirus (FCoV) virulent strain from Aju-92 (cheetah) is in green. Each sequence is labeled as follows: source farm (W, Weller Farm; F, Frederick Animal Shelter; S, Seymour Farm; M, Mount Airy Shelter; A, Ambrose Farm), 4-digit cat identification number, tissue source (fe, feces; af, ascites fluid; co, colon; li, liver; sp, spleen; in, intestine; je, jejunum; ln, lymph node), 2-digit year (e.g., 04 = 2004), and number of clones for each sequence. Scale bar indicates number of substitutions per site.

Appendix Figure 3 Feline coronavirus (FCoV) sequence from cats from the Weller farm. Maximum likelihood phylogenetic tree of unique membrane and nonstructural protein 7b (NSP 7b) FCoV gene sequences showing monophyly correlating to disease status. Cloned sequences from feline infectious peritonitis virus (FIPV) biotypes are shown in red; feline enteric coronavirus (FECV) biotypes are shown in green. A) membrane (maximum likelihood [ML] -ln L = 2646.84352 best tree found by maximum parsimony (MP): length = 270, CI = 0.789, retention index [RI] = 0.971; mid-point rooted); B) NSP 7b (ML -n L= 3997.98885 best tree found by MP: length = 411, confidence interval [CI] = 0.791, RI = 0.981; mid-point rooted). The number of cats is indicated in parenthesis in the key. Each sequence is labeled as follows: 4-digit cat identification number, tissue source (fe, feces; af, ascites fluid; co, colon; li, liver; sp, spleen; in, intestine; je, jejunum; ln, lymph node), 2-digit year (e.g., 04 = 2004), and the unique 3-4-digit sequence number. The number of clones for each sequence is indicated after the sequence label in parenthesis. Where ML tree was congruent with MP tree, branch lengths are indicated below branches; the number of homoplasies is in parenthesis after the branch length. Bootstrap values are shown (MP/minimum evolution/ML) above branches. Virus sequence obtained from cat 4590 in May 2004 and at the time of death due to FIPV in December 2004 is indicated by box. The 2 distinct virus genotypes isolated from this case pre- and postdisease in both the membrane and NSP 7b genes are consistent with the dual circulating virulent and avirulent strains in FCoV pathogenesis. Scale bars indicate substitutions/site.

## References

[1] Addie DD. Clustering of feline coronaviruses in multicat households. Vet J. 2000;159:8–9. 10.1053/tvjl.1999.0429 10640407 PMC7172047

[2] Addie DD, Jarrett O. A study of naturally occurring feline coronavirus infections in kittens. Vet Rec. 1992;130:133–7. 1313617 10.1136/vr.130.7.133

[3] Kennedy M, Citino S, McNabb AH, Moffatt AS, Gertz K, Kania S. Detection of feline coronavirus in captive *Felidae* in the USA. J Vet Diagn Invest. 2002;14:520–2. 12423039 10.1177/104063870201400615

[4] Pedersen NC. A review of feline infectious peritonitis virus infection: 1963–2008. J Feline Med Surg. 2009;11:225–58. 10.1016/j.jfms.2008.09.008 19254859 PMC7129802

[5] Foley JE, Poland A, Carlson J, Pedersen NC. Risk factors for feline infectious peritonitis among cats in multiple-cat environments with endemic feline enteric coronavirus. J Am Vet Med Assoc. 1997;210:1313–8. 9143536

[6] Pedersen NC, Evermann JF, McKeirnan AJ, Ott RL. Pathogenicity studies of feline coronavirus isolates 79-1146 and 79-1683. Am J Vet Res. 1984;45:2580–5. 6084432

[7] de Groot RJ. Feline infectous peritonitis. In: Siddell SG, editor. The *Coronoviridae*. New York: Plenum Press; 1995. p. 293–309.

[8] Weiss RC, Scott FW. Pathogenesis of feline infectious peritonitis: nature and development of viremia. Am J Vet Res. 1981;42:382–90. 6267961

[9] Kipar A, Kohler K, Leukert W, Reinacher M. A comparison of lymphatic tissues from cats with spontaneous feline infectious peritonitis (FIP), cats with FIP virus infection but no FIP, and cats with no infection. J Comp Pathol. 2001;125:182–91. 10.1053/jcpa.2001.0501 11578135

[10] Kipar A, Meli ML, Failing K, Euler T, Gomes-Keller MA, Schwartz D, Natural feline coronavirus infection: differences in cytokine patterns in association with the outcome of infection. Vet Immunol Immunopathol. 2006;112:141–55. 10.1016/j.vetimm.2006.02.004 16621029 PMC7112699

[11] Hunziker L, Recher M, Macpherson AJ, Ciurea A, Freigang S, Hengartner H, Hypergammaglobulinemia and autoantibody induction mechanisms in viral infections. Nat Immunol. 2003;4:343–9. 10.1038/ni911 12627229

[12] Poland AM, Vennema H, Foley JE, Pedersen NC. Two related strains of feline infectious peritonitis virus isolated from immunocompromised cats infected with a feline enteric coronavirus. J Clin Microbiol. 1996;34:3180–4. 8940468 10.1128/jcm.34.12.3180-3184.1996 PMC229479

[13] Vennema H, Poland A, Foley J, Pedersen NC. Feline infectious peritonitis viruses arise by mutation from endemic feline enteric coronaviruses. Virology. 1998;243:150–7. 10.1006/viro.1998.9045 9527924 PMC7131759

[14] Rottier PJ, Nakamura K, Schellen P, Volders H, Haijema BJ. Acquisition of macrophage tropism during the pathogenesis of feline infectious peritonitis is determined by mutations in the feline coronavirus spike protein. J Virol. 2005;79:14122–30. 10.1128/JVI.79.22.14122-14130.2005 16254347 PMC1280227

[15] Stoddart CA, Scott FW. Intrinsic resistance of feline peritoneal macrophages to coronavirus infection correlates with in vivo virulence. J Virol. 1989;63:436–40. 2521188 10.1128/jvi.63.1.436-440.1989 PMC247703

[16] Haijema BJ, Volders H, Rottier PJ. Switching species tropism: an effective way to manipulate the feline coronavirus genome. J Virol. 2003;77:4528–38. 10.1128/JVI.77.8.4528-4538.2003 12663759 PMC152114

[17] Can-Sahna K, Soydal Ataseven V, Pinar D, Oguzoglu TC. The detection of feline coronaviruses in blood samples from cats by mRNA RT-PCR. J Feline Med Surg. 2007;9:369–72. 10.1016/j.jfms.2007.03.002 17478116 PMC7128869

[18] Dye C, Siddell SG. Genomic RNA sequence of feline coronavirus strain FCoV C1Je. J Feline Med Surg. 2007;9:202–13. 10.1016/j.jfms.2006.12.002 17363313 PMC2582377

[19] Hartley O, Klasse PJ, Sattentau QJ, Moore JP. V3: HIV's switch-hitter. AIDS Res Hum Retroviruses. 2005;21:171–89. 10.1089/aid.2005.21.171 15725757

[20] Ballesteros ML, Sanchez CM, Enjuanes L. Two amino acid changes at the N-terminus of transmissible gastroenteritis coronavirus spike protein result in the loss of enteric tropism. Virology. 1997;227:378–88. 10.1006/viro.1996.8344 9018137 PMC7130969

[21] Saif LJaS. K. Transmissible gastroenteritis virus and porcine respiratory coronavirus. In: Zimmerman JJ, editor. Diseases of swine. 9th ed. Ames (IA): Iowa State University Press; 2006. p. 489–516.

[22] Sanchez CM, Izeta A, Sanchez-Morgado JM, Alonso S, Sola I, Balasch M, Targeted recombination demonstrates that the spike gene of transmissible gastroenteritis coronavirus is a determinant of its enteric tropism and virulence. J Virol. 1999;73:7607–18. 10438851 10.1128/jvi.73.9.7607-7618.1999 PMC104288

[23] Mongkolsapaya J, Dejnirattisai W, Xu XN, Vasanawathana S, Tangthawornchaikul N, Chairunsri A, Original antigenic sin and apoptosis in the pathogenesis of dengue hemorrhagic fever. Nat Med. 2003;9:921–7. 10.1038/nm887 12808447

[24] Anishchenko M, Bowen RA, Paessler S, Austgen L, Greene IP, Weaver SC. Venezuelan encephalitis emergence mediated by a phylogenetically predicted viral mutation. Proc Natl Acad Sci U S A. 2006;103:4994–9. 10.1073/pnas.0509961103 16549790 PMC1458783

[25] Thompson JD, Gibson TJ, Plewniak F, Jeanmougin F, Higgins DG. The CLUSTAL_X windows interface: flexible strategies for multiple sequence alignment aided by quality analysis tools. Nucleic Acids Res. 1997;25:4876–82. 10.1093/nar/25.24.4876 9396791 PMC147148

[26] Maddison DRaM. W.P. MacClade 3.05. Sunderland (MA): Sinauer; 1995.

[27] Swofford DL. PAUP*: Phylogenetic Analysis Using Parsimony (*and other methods). Sunderland (MA): Sinauer; 2002.

[28] Posada D, Crandall KA. MODELTEST: testing the model of DNA substitution. Bioinformatics. 1998;14:817–8. 10.1093/bioinformatics/14.9.817 9918953

[29] Kumar S, Tamura K, Nei M. MEGA3: Integrated software for Molecular Evolutionary Genetics Analysis and sequence alignment. Brief Bioinform. 2004;5:150–63. 10.1093/bib/5.2.150 15260895

[30] Pearks Wilkerson AJ, Teeling EC, Troyer JL, Bar-Gal GK, Roelke M, Marker L, Coronavirus outbreak in cheetahs: lessons for SARS. Curr Biol. 2004;14:R227–8. 10.1016/j.cub.2004.02.051 15043830 PMC7126726

[31] Rottier PJ. The coronavirus membrane glycoprotein. In: Siddell SG, editor. The *Coronaviridae*. New York: Plenum Press; 1995. p. 115–40.

[32] He Y, Zhou Y, Siddiqui P, Niu J, Jiang S. Identification of immunodominant epitopes on the membrane protein of the severe acute respiratory syndrome-associated coronavirus. J Clin Microbiol. 2005;43:3718–26. 10.1128/JCM.43.8.3718-3726.2005 16081901 PMC1234014

[33] Heeney JL, Evermann JF, McKeirnan AJ, Marker-Kraus L, Roelke ME, Bush M, Prevalence and implications of feline coronavirus infections of captive and free-ranging cheetahs (*Acinonyx jubatus*). J Virol. 1990;64:1964–72. 2157864 10.1128/jvi.64.5.1964-1972.1990 PMC249350

[34] Pontius JU, Mullikin JC, Smith DR, Lindblad-Toh K, Gnerre S, Clamp M, Initial sequence and comparative analysis of the cat genome. Genome Res. 2007;17:1675–89. 10.1101/gr.6380007 17975172 PMC2045150

