# Supplementary material for: Genetics and Pathogenesis of Feline Infectious Peritonitis Virus
Source: Emerg Infect Dis. 2009 Sep;15(9):1445–52. doi: 10.3201/eid1509.081573 (PMC2819880; doi:10.3201/eid1509.081573)
Supplement: Appendix Table 1 — Clinical, demographic, and FCoV viral RT-PCR success data from 56 domestic cats sampled in Maryland, USA, 2004-2006* [file 08-1573_appT1-s1.pdf]

Publisher: CDC; Journal: Emerging Infectious Diseases
Article Type: Research; Volume: 15; Issue: 9; Year: 2009; Article ID: 08-1573
DOI: 10.3201/eid1509.081573; TOC Head: Research

Appendix Table 1. Clinical, demographic, and FCoV viral RT-PCR success data from 56 domestic cats sampled in Maryland, USA, 2004–2006*

			FCoV gene sequences						
Cat ID	Farm	Sex/age	2004	2005	2006	Status 2007	IHC/Histo	PE	FCoV
4549	Weller	M/1.5 y	M,7,P,3	–	–	D	Pos	–	Pos
4561	FCAC	F/3 y	M,P,3	–	–	E	Neg (pos SI)	WNL	Neg
4562	Palmer	M/1 y	–	–	–	E	Neg	WNL	Neg
4563	Palmer	M/1 y	–	–	–	E	Neg	WNL	Pos
4564	Palmer	M/1 y	–	–	–	E	Neg	WNL	Neg
4566	Weller	M/1.5 y	M,7,P,3	–	–	D	Pos	AB	Pos
4580	Weller	F/1 y	–	–	–	H	–	WNL	Pos
4581	Weller	F/1 y	7	–	–	H	–	WNL	Pos
4582	Weller	F/1 y	M	M	–	H	–	WNL	Pos
4583	Weller	M/1 y	–	M	7	H	–	WNL	–
4584	Weller	F/2 y	7	–	3	H	–	WNL	Pos
4585	Weller	M/1 y	M	–	M	H	–	WNL	Pos
4586	Weller	F/1 y	M,7,3	–	–	H	–	WNL	Pos
4587	Weller	M/1 y	–	–	–	H	–	WNL	Pos
4588	Weller	M/1 y	M	–	–	H	–	WNL	Pos
4589	Weller	M/1 y	M,7	–	–	H	–	WNL	Pos
4590	Weller	M/2 y	M,7	M,7,3	–	D	Pos	AB	Pos
4591	Weller	F/1 y	M,7,P	M	–	H	–	WNL	Pos
4592	Weller	F/1 y	–	M	–	H	–	WNL	Pos
4593	Weller	M/1 y	7,P	M	M	H	–	WNL	Pos
4594	Weller	F/1 y	M,7,P	–	M,7	H	–	WNL	Pos
4595	Weller	F/1 y	M,7,P,3	–	–	H	–	WNL	Pos
4596	Weller	F/1 y	–	–	–	H	–	AB	Pos
4597	Weller	F/1 y	P	–	M,7,3	H	–	WNL	Pos
4606	Weller	F/4 y	M	–	M,7	H	–	WNL	Pos
4607	Weller	M/3 y	–	–	–	H	–	WNL	Pos
4608	Weller	F/3 y	–	–	–	H	–	WNL	Neg
4609	Weller	F/1 y	7	–	–	H	–	AB	Pos
4611	Weller	M/7 y	–	–	–	H	–	WNL	Pos
4612	Weller	F/1 y	7,3	–	–	H	–	WNL	Pos
4613	Weller	F/4 y	–	–	–	H	–	WNL	Pos
4614	Weller	M/5 y	–	–	–	H	–	WNL	Pos
4615	Weller	F/7 y	–	–	–	H	–	WNL	Pos
4616	Weller	F/6 y	–	–	–	H	–	WNL	Pos
4618	Weller	M/1 y	M,7	–	–	D	Pos	AB	Pos
4620	Weller	M/6 y	–	–	–	D	Pancreatitis	AB	Neg
4623	Weller	UK/UK	–	–	–	D	Neg	AB	Neg
4624	Seymour	F/2y	–	M,P,3,7	–	E	Lymphoma	AB	–
4625	Weller	F/1 y	P	–	–	D	Lymphoma	AB	–
4626	Ambrose	F/11 y	–	–	–	H	–	WNL	–
4627	Ambrose	M/11 y	–	–	–	H	–	WNL	Pos
4628	Ambrose	F/7 y	–	–	–	H	–	WNL	Pos
4629	Ambrose	F/7 y	–	–	–	H	–	WNL	Pos
4630	Ambrose	M/4 y	–	–	–	H	–	WNL	Pos
4631	Ambrose	M/2 y	–	–	–	H	–	WNL	Pos
4653	Ambrose	F/4 y	–	–	M,7,P,3	D	Pos	AB	Pos
4654	NM	M/2 mo	–	–	–	D	SI enteritis	AB	Pos
4655	FCAC	F/2 mo	–	–	–	E	Neg	AB	Pos
4656	FCAC	M/6 wk	–	7,M,3	–	E	Neg	WNL	–
4657	FCAC	M/6 wk	–	M,7	–	E	Neg	WNL	Pos
4658	FCAC	M/3 wk	–	–	–	E	Neg	WNL	Pos
4659	FCAC	M/6 wk	–	M,7,3	–	E	Neg	WNL	Pos
4660	FCAC	M/8 wk	–	–	–	E	Neg	WNL	Pos
4662	Weller	M/4 mo	–	–	M,7,P,3	E	Pos	–	–
4663	Weller	F/4 mo	–	–	M,7,P,3	E	Pos	–	–
4664	NM	M/6 mo	–	–	M,7,3	E	Pos	–	–

*FCoV, feline coronavirus; RT-PCR, reverse transcription–PCR; ID, identification number; IHC, immunohistochemical; PE, physical exam; M, membrane; D, dead; Pos, positive; FCAC, Frederick County Animal Shelter; E, euthanized; Neg, negative; SI, small intestine; WNL, within normal limits; AB, abnormal pathology; H, healthy; UK, unknown; NM, New Market Animal Shelter. Shaded cells in left column represent 8 FIP cases.
