# Supplementary material for: Genetics and Pathogenesis of Feline Infectious Peritonitis Virus
Source: Emerg Infect Dis. 2009 Sep;15(9):1445–52. doi: 10.3201/eid1509.081573 (PMC2819880; doi:10.3201/eid1509.081573)
Supplement: Appendix Table 2 — Genotype composites of 8 FIPV and 19 FECV domestic cats in Maryland, USA, sampled 25 times at 5 variable amino acids in the FCoV membrane protein* [file 08-1573_appT2-s2.pdf]

Publisher: CDC; Journal: Emerging Infectious Diseases
Article Type: Research; Volume: 15; Issue: 9; Year: 2009; Article ID: 08-1573
DOI: 10.3201/eid1509.081573; TOC Head: Research

Appendix Table 2. Genotype composites of 8 FIPV and 19 FECV domestic cats in Maryland, USA, sampled 25 times at 5 variable amino acids in the FCoV membrane protein*

Cloned NT sequences	Amino acid sequences	FCA cat ID†	Source‡	108§	120§	138§	163§	199§	Year	Farm	Genotype
17	10	4549	AF, LI, LN, CO	Y	I	V	A	L	2004	Weller	I
18	7	4566	JE	–	–	–	–	–	2004	Weller	I
17	12	4618	LI, LN, FE	–	–	–	–	–	2004	Weller	I
3	3	4662	FE	–	–	–	–	–	2006	Weller	I
13	5	4663	FE, LI	–	–	–	–	–	2006	Weller	I
3	2	4664	JE	–	–	–	–	–	2006	MA	I
12	1	4590	SI	–	–	–	–	–	2004 Dec	Weller	I
6	3	4653	SP, LI	–	–	I	–	–	2004	Ambrose	II
3	3	4662	LI	–	–	I	–	–	2006	Weller	II
11	7	4664	AF, LI	–	–	I	–	–	2006	MA	II
5	2	4590	FE	H	–	I	V	I	2004 May	Weller	III
13	2	4582	FE	H	–	I	V	I	2004	Weller	III
6	2	4583	FE	H	–	I	V	I	2005	Weller	III
3	6	4585	FE	H	–	I	V	I	2004	Weller	III
9	3	4585-06	FE	H	–	I	V	I	2006	Weller	III
2	3	4586	FE	H	–	I	V	I	2004	Weller	III
5	3	4588	FE	H	–	I	V	I	2004	Weller	III
15	1	4591	FE	H	–	I	V	I	2004	Weller	III
6	2	4594-06	FE	H	–	I	V	I	2006	Weller	III
2	1	4595	FE	H	–	I	V	I	2004	Weller	III
6	4	4597	FE	H	–	I	V	I	2006	Weller	III
3	2	4606	FE	H	–	I	V	I	2004	Weller	III
1	1	4593	FE	H	–	I	V	–	2004	Weller	IV
2	2	4606-06	FE	–	V	I	–	–	2006	Weller	V
9	4	4561	FE	–	V	I	–	–	2004	FCAC	V
6	2	4582-06	FE	–	V	I	–	–	2006	Weller	V
3	1	4591-05	FE	–	V	I	–	–	2005	Weller	V
3	2	4592	FE	–	V	I	–	–	2005	Weller	V
7	6	4593-06	FE	–	V	I	–	–	2006	Weller	V
10	3	4656	FE	–	V	I	–	–	2005	FCAC	V
9	9	4659	FE	–	V	I	–	–	2005	FCAC	V
1	1	4655	FE	–	V	–	–	–	2005	FCAC	VI
6	2	4594	FE	–	–	–	–	–	2004	Weller	VI
6	4	4624	FE	–	–	–	–	–	2005	Seymour	VI
6	5	4657	FE	–	–	–	–	–	2005	FCAC	VI
8	8	Aju-92	LI	–	V	–	–	–	1982	WSP	VI
–	–	FIPV79-1146	GenBank	–	V	–	–	–	1991	Wash	VI
–	–	FIPV79-1683	GenBank	–	V	I	–	–	1991	Wash	V
–	–	SARS	GenBank	–	–	F	L	C	2003	–	–
–	–	MHV-1	GenBank	–	–	I	M	F	2007	–	–
–	–	IBV-Beu	GenBank	P	–	I	T	C	2001	–	–
–	–	BCV-K	GenBank	–	–	I	M	Y	2007	–	–
–	–	HCV-229E	GenBank	W	F	F	V	V	1990	–	–
–	–	TGEV	GenBank	–	–	I	–	M	1986	–	–

*FIPV, feline infectious peritonitis virus; FECV, chronic feline enteric infection; FCoV, feline coronavirus; NT, nucleotide; AF, ascites fluid; LI, liver; LN, lymph note; CO, colon; JE, jejunem; FE, feces; SI, small intestine; SP, spleen; FCAC, Frederick County Animal Shelter.

†No. corresponding unique amino acid sequences. Pink shading, FIPV cats; green shading, FECV cats; blue shading, FCoV-infected healthy cats; gold shading, FCoV-Aju cat.

‡Identification number (see Appendix Table 1).

§Membrane protein amino acid residues tyrosine (Y), isoleucine (I), valine (V), alanine (A), leucine (L), histidine (H) (GenBank reference sequence number P04135). –, identical amino acid residue as reference (first line); grey shading indicates different amino acid. Genotype composite of FCoV-Aju reported here; cell line (6) and reference sequences for SARS-CoV, MHV-1, IBV-Beu, BVC-K, HcoV-229E, TGEV-Purdue, and FCoV 79-1146 (GenBank accession numbers P59596, AB587268, P69602, BAF75636, P15422, PO4135, and P25878, respectively) are also shown.
