# Supplementary material for: Genetics and Pathogenesis of Feline Infectious Peritonitis Virus
Source: Emerg Infect Dis. 2009 Sep;15(9):1445–52. doi: 10.3201/eid1509.081573 (PMC2819880; doi:10.3201/eid1509.081573)
Supplement: Appendix Table 3 — Additional clinical data from 56 domestic cats sampled in Maryland, USA, 2004-2006* [file 08-1573_appT3-s3.pdf]

Publisher: CDC; Journal: Emerging Infectious Diseases
Article Type: Research; Volume: 15; Issue: 9; Year: 2009; Article ID: 08-1573
DOI: 10.3201/eid1509.081573; TOC Head: Research

Appendix Table 3. Additional clinical data from 56 domestic cats sampled in Maryland, USA, 2004–2006*

Cat ID	Name	Hem	RBC	WBC	Lymph	Neut	Bands	Pro	Glo	Bili	Toxo/ FeLV	FIV
FCA-4549	Hank	41.1	8.55	4.2	210	3,864	84	–	–	–	Toxo 1:128	Neg
FCA-4561	PregnantF	24.9	5.79	5.1	4,539	357	0	–	–	–	–	Neg
FCA-4562	Bryan	0.3	0.23	8.3	415	387	0	7.4	3.6	0.3	–	Neg
FCA-4563	Dreamcylcle	27.3	6.32	9.4	1,222	7,802	0	7.7	4.4	0.1	Neg	Neg
FCA-4564	Michael	7.1	0.24	6.5	975	810	0	7.2	3.6	0.1	Neg	Neg
FCA-4566	Ace	21.3	4.68	5.9	295	5,192	0	–	–	–	–	–
FCA-4580	Phoebe	33.6	7.61	11.2	5,936	3,920	0	–	–	–	–	–
FCA-4581	Palmer	42.7	9.89	13.8	4,002	8,142	0	–	–	–	–	–
FCA-4582	Ying	40.7	8.6	12.2	5,490	4,880	0	–	–	–	–	–
FCA-4583	Yang	–	–	–	–	–	–	–	–	–	–	–
FCA-4584	Sydney	28.9	6.12	8.2	1,476	6,150	0	–	–	–	–	–
FCA-4585	Swain	–	–	–	–	–	–	–	–	–	–	–
FCA-4586	Josie	36.6	8.05	11	2,970	6,050	0	–	–	–	–	–
FCA-4587	Mo jo	41.3	8.33	11.8	3,068	7,080	0	–	–	–	–	–
FCA-4588	Simpson	36.5	8.36	7.7	2,079	5,005	0	–	–	–	–	–
FCA-4589	Ty	41.3	9.58	10.3	3,193	5,871	0	–	–	–	–	–
FCA-4590	Diesel	–	–	–	–	–	–	–	–	–	Neg/neg	Neg
FCA-4591	Chenille	38	8.39	18	9,180	7,200	0	–	–	–	–	–
FCA-4592	Kerrigan	39.8	8.85	19.4	9,312	8,730	0	–	–	–	–	–
FCA-4593	Leroy	39.2	8.75	11.4	2,508	7,524	0	–	–	–	–	–
FCA-4594	Ursa	–	–	–	–	–	–	–	–	–	–	–
FCA-4595	Snuffy	–	–	–	–	–	–	–	–	–	–	–
FCA-4596	Teva	–	–	–	–	–	–	–	–	–	–	–
FCA-4597	Bings	27.9	5.95	14.7	2,058	10,731	0	–	–	–	–	–
FCA-4606	Elsa	34.1	7.4	15.4	3,850	9,856	0	–	–	–	–	–
FCA-4607	BamBam	32.3	7.54	12.4	3,720	8,556	0	–	–	–	–	–
FCA-4608	Layla	32.8	7.68	14.9	4,619	8,940	–	–	–	–	–	–
FCA-4609	Queenie	33.7	6.61	6.9	2,208	3,933	0	–	–	–	–	–
FCA-4611	Cheeks	35.4	7.61	14.6	3,212	10,366	0	–	–	–	–	–
FCA-4612	Rocket	29.6	6.12	10.1	2,323	6,868	0	–	–	–	–	–
FCA-4613	Jasmine	40	7.83	11.2	2,240	7,280	0	–	–	–	–	–
FCA-4614	BoBo	37	9.39	7	1,260	5,180	0	–	–	–	–	–
FCA-4615	Patches	30.4	6.64	35	1,750	29,750	1,400	–	–	–	–	–
FCA-4616	Isis	36.1	6.54	15.2	4,104	8,816	0	–	–	–	–	–
FCA-4618	Elliot	–	–	–	–	–	–	–	–	–	–	–
FCA-4620	Tony	–	–	–	–	–	–	–	–	–	Neg/neg	Neg
FCA-4623	KofBings	–	–	–	–	–	–	–	–	–	–	–
FCA-4624	Zoe	30.5	6.46	26	16,900	6,760	260	6.8	3.4	0.1	–	–
FCA-4625	Goldie	–	–	–	–	–	–	–	–	–	FeLV neg	Neg
FCA-4626	Saddie	–	–	–	–	–	–	–	–	–	FeLV pos	Neg
FCA-4627	Sassy	31.6	7.93	8	6,000	1,600	0	–	–	–	–	–
FCA-4628	Rosie	qns	qns	8	2,560	4,880	0	7.5	5.1	0.3	Neg/neg	Neg
FCA-4629	Lilly	18.8	4.93	7.4	962	5,994	0	–	–	–	Neg/neg	Neg
FCA-4630	Rudy	32.9	7.2	10.3	6,901	2,781	0	7.2	4.2	0.3	Neg/neg	Neg
FCA-4631	Boy	qns	qns	5.6	3,136	2,184	0	–	–	–	–	–
FCA-4653	Zoe	28.5	7.4	18.6	2,046	15,996	0	9.4	6.9	0.1	Neg/neg	Neg
FCA-4654	Thkitten	–	–	–	–	–	–	–	–	–	–	–
FCA-4655	Penny	7	4.62	6.6	2,178	4,158	0	–	–	–	–	–
FCA-4656	Basil	–	–	–	–	–	–	–	–	–	–	–
FCA-4657	Parsley	–	–	–	–	–	–	–	–	–	–	–
FCA-4658	FCAC	–	–	–	–	–	–	–	–	–	–	–
FCA-4659	Concerta	–	–	–	–	–	–	–	–	–	–	–
FCA-4660	Starfox	–	–	–	–	–	–	–	–	–	–	–
FCA-4662	OJ male	–	–	–	–	–	–	–	–	–	–	–
FCA-4663	OJ female	–	–	–	–	–	–	–	–	–	–	–
FCA-4664	Buster	–	–	–	–	–	–	–	–	–	–	–

*ID, identification number; hem, hematocrit (%); RBC, red blood cells; WBC, white blood cells; lymph, lymphocytes; neut, neutrophils segmented; bands, neutrophils bands; pro, total protein (g/dL); Glo, globulin (g/dL); Bil, total bilirubin (mg/dL); Toxo, *Toxoplasma gondii*; FeLV, feline leukemia virus; FIV, feline immunodeficiency virus; –, not done; neg, negative; qns, quantity of sample was insufficient for test; pos, positive; FCAC, Frederick County Animal Shelter. Gray shading represents feline infectious peritonitis virus cases.
