# Supplementary material for: Genetics and Pathogenesis of Feline Infectious Peritonitis Virus
Source: Emerg Infect Dis. 2009 Sep;15(9):1445–52. doi: 10.3201/eid1509.081573 (PMC2819880; doi:10.3201/eid1509.081573)
Supplement: Appendix Figure 1 — Alignment of variable sites of unique amino acid sequences of membrane and nonstructural protein 7b (NSP 7b) genes from feline infectious peritonitis virus (FIPV) cases (gray shaded), from feline enteric coronavirus (FECV) asymptomatic cats, from feline coronavirus (FCoV)-Aju, and reference sequences for severe acute respiratory syndrome coronavirus (CoV), MHV-1, IBV-Beu, BVC-K, HcoV-229E, TGEV-Purdue, and FCoV 79-1146 (GenBank accession nos. P59596, AB587268, P69602, BAF75636, P15422, PO4135, and P25878, respectively). FCoV reference sequences for FECVUCD, FIPVUCD1, FIPV791146, and FIPVUCD3 are also included. Diagnostic sites are highlighted in the membrane. For membrane, cat ID and 2-digit year of sampling is listed and the number of original clones is in parenthesis; the frequency of unique amino acid sequences is reported in column 2. No diagnostic sites were found correlating with FIPV and FECV biotype in NSP 7b. [file 08-1573_appF1-s4.pdf]

	aa	LGD ID		4	6	8	9	12	14	15	16	18	20	21	25	28	29	33	35	36	37	39	43	44	45	48	49	51	52	53	54	57	59	60	63	65	66	68	73	74	75	76	77	78	81	82	85	86	88	90	93	94	95	96	100	102	105	111	112	119	122	123	126	129	130	132	134	136	139	143	145	147
4549-04 (17)	8	4549afFD07	P	Q	S	L	V	I	M	L	I	W	F	W	L	T	I	Y	E	Y	Q	S	M	F	G	I	A	A	V	V	T	L	M	M	V	S	V	L	K	S	W	W	S	F	E	T	I	L	V	A	R	S	Y	V	D	T	G	L	S	G	M	A	L	E	H	P	Y	M	T	T	V	T
	1	4549afFE07	.	.	.	.	.	.	.	.	.	.	L	.	.	.	.	.	.	.	.	.	.	.	.	.	.	.	.	.	.	.	.	.	.	.	.	.	.	.	.	.	.	.	.	.	.	.	.	.	.	.	.	.	.	.	.	.	.	S	.	.	.	.	.	.	.	.	.	.	.	.
	1	4549liFD11	.	.	.	.	.	.	.	.	.	.	.	.	.	.	.	.	.	.	.	.	.	.	.	.	.	.	.	.	.	.	.	.	.	.	.	.	.	.	.	.	P	.	.	.	.	.	.	.	.	.	.	.	.	.	.	.	.	.	.	.	.	.	.	.	.	.	.	.	.	.
	5	4549coFB01	L	.	.	.	.	.	.	.	.	.	.	.	.	.	.	.	.	.	.	.	.	.	.	.	.	.	.	.	.	.	.	.	.	.	.	.	.	.	.	.	.	.	.	.	.	.	.	.	.	.	.	.	.	.	.	.	.	.	.	.	.	.	.	.	.	.	.	.	.	.
	1	4549coFG01	.	.	.	.	.	.	.	.	.	.	L	.	.	.	.	.	.	.	.	.	.	.	.	.	.	.	.	.	.	.	.	.	.	.	.	.	.	.	.	.	.	.	.	.	.	.	.	.	.	.	.	.	.	.	.	.	.	S	.	.	.	.	.	.	.	.	.	.	.	.
	1	I4549lnFA03	.	.	.	.	.	.	.	.	.	.	.	.	.	.	.	.	.	.	.	.	.	.	.	.	.	.	.	.	.	.	.	.	.	.	.	.	.	.	.	.	.	.	.	.	.	.	.	.	.	.	.	.	.	.	.	.	.	.	.	.	.	.	.	.	.	.	.	.	.	.
4566-04 (18)	4	B4566in2FF02	.	.	.	.	.	.	.	.	.	.	L	.	.	.	.	.	.	.	.	.	.	.	.	.	.	.	.	.	.	.	.	.	.	.	.	.	.	.	.	.	.	.	.	.	.	.	.	.	.	.	.	.	.	.	.	.	.	.	.	.	.	.	.	.	.	.	K	.	.	.
	10	B4566in2FB02	.	.	.	.	.	.	.	.	.	.	L	.	.	.	.	.	.	.	.	.	.	.	.	.	.	.	.	.	.	.	.	.	.	.	.	.	.	.	.	.	.	.	.	.	.	.	.	.	.	.	.	.	.	.	.	.	.	.	.	.	.	.	.	.	.	.	.	.	.	.
	1	B4566in2FA02	.	.	.	.	.	.	.	.	.	.	L	.	.	.	.	.	.	.	.	.	.	.	.	.	.	.	.	.	.	P	.	.	.	.	.	.	.	.	.	.	.	.	.	.	.	.	.	.	.	.	.	.	.	.	.	.	.	.	.	.	.	.	.	.	.	.	.	.	.	.
	3	A4566inFD06	.	.	.	.	.	.	.	.	.	.	L	.	.	.	.	.	.	.	.	.	.	.	.	.	.	.	.	.	.	.	.	.	.	.	.	.	.	.	.	.	.	.	.	.	.	.	.	.	.	.	.	.	.	.	.	.	.	.	.	.	.	.	.	.	.	.	.	.	.	A
4618-04 (15)	2	4618liFD08	.	.	.	.	.	.	.	.	.	.	L	.	.	.	.	.	.	.	.	.	.	.	.	.	.	.	.	.	.	.	.	.	.	.	.	.	.	.	.	.	.	.	.	.	.	.	.	.	.	.	.	.	.	.	.	.	.	.	.	.	.	.	.	.	.	.	.	.	.	.
	1	4618liFB08	.	.	.	.	.	.	.	.	.	.	L	.	.	.	.	.	.	.	.	.	.	.	.	.	.	.	.	.	S	.	.	.	.	.	.	.	.	.	.	.	.	.	.	.	.	.	.	.	.	.	.	.	.	.	S	.	.	.	.	.	.	.	.	.	.	.	.	.	.	.
	1	4618liFF04	.	.	.	.	.	.	.	.	.	.	L	R	.	.	.	.	.	.	.	P	.	.	.	.	.	.	.	.	.	.	.	T	.	.	.	.	.	.	.	.	.	.	.	.	.	.	.	.	.	.	.	.	.	.	.	.	.	.	.	.	.	.	.	.	.	.	.	.	.	.
	1	4618feFC08	.	.	.	.	.	.	.	.	.	.	L	.	.	.	.	.	.	.	.	P	.	.	.	.	.	.	.	.	.	.	.	.	.	.	.	.	.	.	.	.	.	.	.	.	.	.	L	.	.	.	.	.	G	.	.	.	.	.	.	.	.	.	.	.	.	.	.	.	.	.
	2	4618feFD08	.	.	.	.	.	.	.	.	.	.	L	.	.	.	.	.	.	.	.	P	.	.	.	.	.	.	.	.	.	.	.	.	.	.	.	.	.	.	.	.	.	.	.	.	.	.	.	.	.	.	.	.	.	.	.	.	.	.	.	.	.	.	.	.	.	.	.	.	.	.
	1	4618feFE08	.	.	.	.	.	.	.	.	.	.	L	.	.	.	.	.	.	.	.	P	.	.	.	.	.	.	.	.	.	.	.	.	.	.	.	.	.	.	.	.	.	.	.	.	.	.	L	.	.	.	.	.	.	.	.	.	.	.	.	.	.	.	.	.	.	.	.	.	.	.
	2	4618feFG08	.	.	.	.	.	.	.	.	.	.	L	.	.	.	.	.	.	.	.	P	.	.	.	.	.	.	.	.	.	.	.	.	.	.	.	.	.	.	.	.	.	.	.	.	.	.	.	.	.	.	C	.	.	.	.	.	.	.	.	.	.	.	.	.	.	.	.	.	.	.
	1	4618feFA08bgr12	.	.	.	.	.	.	.	.	.	.	L	.	.	.	.	.	.	.	.	P	.	.	.	.	.	.	.	.	.	.	.	.	.	.	.	.	.	.	.	.	.	.	.	.	.	.	.	.	.	.	.	.	.	.	.	.	.	.	L	.	.	.	.	.	.	.	.	.	.	.
	1	4618lnc2	.	.	.	.	.	.	.	.	.	.	L	.	.	.	.	.	.	.	.	P	.	.	S	.	.	.	.	I	.	.	.	.	.	.	.	.	.	.	.	.	.	.	.	.	.	.	.	.	.	.	.	.	.	.	.	.	.	C	.	.	.	.	.	.	.	.	.	.	.	.
	1	4618lnFC03	.	.	.	.	.	.	.	.	.	.	L	R	.	.	.	.	.	.	.	P	.	.	.	.	.	.	.	.	.	.	.	.	.	.	F	.	.	.	.	.	.	.	.	.	.	.	.	.	.	.	.	.	.	.	.	.	.	.	.	.	.	.	.	.	.	.	.	.	.	.
4590 si(1)	1	4618lnFA03	.	.	.	.	.	.	.	.	.	.	L	R	.	.	.	.	.	.	.	P	.	.	.	.	.	.	.	.	.	.	.	.	.	.	.	.	.	.	.	.	.	.	.	.	.	.	.	.	.	.	.	.	.	.	.	.	.	.	.	.	.	.	.	.	.	.	.	.	.	.
	1	4618lnFH03	.	.	.	.	.	.	.	.	.	.	L	R	.	.	.	.	.	.	.	P	.	.	.	.	.	.	.	.	.	.	.	.	.	.	.	.	.	.	.	.	.	.	.	.	.	.	.	.	.	.	.	.	.	.	.	.	.	.	.	.	.	.	.	.	F	.	.	.	.	.
4653-06 (6)	3	4653spFD02b	.	.	.	.	I	.	.	.	.	.	L	.	.	.	.	.	.	.	.	.	L	.	.	.	.	.	.	.	.	.	.	.	.	.	I	.	.	.	.	.	.	.	.	.	.	.	.	.	.	.	.	I	.	N	.	P	.	.	.	.	.	D	.	.	.	.	.	.	.	.
	1	4653spFA02b	.	.	.	.	I	.	.	.	.	.	L	.	.	.	.	.	.	.	.	.	L	.	.	.	.	.	.	.	.	.	.	.	.	.	I	.	.	.	.	.	.	.	.	.	.	.	.	.	.	.	.	I	.	N	.	.	.	.	.	.	.	D	.	.	C	.	.	.	.	.
	2	4653liFF12gr25	.	.	.	.	I	.	.	.	.	.	L	.	.	.	.	.	.	.	.	.	L	.	.	.	.	.	.	.	.	.	.	.	.	.	I	.	.	.	.	.	.	.	.	.	.	.	.	.	.	.	.	I	.	N	.	.	.	.	.	.	.	D	.	.	.	.	.	.	.	.
4662li-06 (3)	1	4662liFB09gr10	.	.	.	.	.	.	.	.	.	.	L	.	I	.	.	.	.	.	.	.	.	.	.	.	.	.	.	.	.	.	.	.	.	A	I	.	.	.	.	.	.	.	.	.	.	.	.	.	.	.	.	.	.	I	.	.	.	.	I	.	.	.	.	.	.	.	.	.	.	.
	1	4662liFB09gr10	.	.	.	.	.	.	.	.	.	.	L	.	I	.	.	.	.	.	.	.	.	.	.	.	.	.	.	.	.	.	.	.	.	A	I	.	.	.	.	.	.	.	.	.	.	.	.	.	.	.	.	.	.	I	.	.	.	.	I	.	.	.	.	.	.	.	.	.	.	.
	1	4662liFC09	.	.	.	.	.	.	.	.	.	.	L	.	.	.	.	.	.	.	.	P	.	.	.	.	.	.	.	.	.	.	.	.	.	A	I	.	.	.	.	.	.	.	.	.	.	.	.	.	.	.	.	.	.	I	.	.	.	.	I	.	.	.	.	.	.	T	.	.	.	.
4662fe-06(9)	5	4662feFH05gr11	.	.	.	.	.	.	.	.	.	.	L	.	.	.	.	.	.	.	.	.	.	.	.	.	.	.	.	.	.	.	.	.	.	.	.	.	.	.	.	.	.	.	.	.	.	.	.	.	.	.	.	.	.	.	.	.	.	.	.	.	.	.	.	.	.	.	.	.	.	.
	3	4662feFF05	.	.	.	.	.	.	.	.	.	R	L	.	.	.	.	.	.	.	.	.	.	.	.	.	.	.	.	.	.	.	.	.	.	.	F	.	.	.	.	.	.	.	.	.	.	.	.	.	.	.	.	.	.	.	.	.	.	.	.	.	.	.	.	.	.	.	.	.	.	.
	1	4662feFA05	.	.	.	.	.	.	.	.	.	.	L	.	.	.	.	.	.	.	.	.	.	.	.	.	.	.	.	.	.	.	.	.	.	.	.	.	.	.	.	.	.	S	.	.	.	.	.	.	.	.	.	.	.	.	.	.	.	.	.	.	.	.	.	.	.	.	.	.	.	.
4663-06 (22)	10	4663feFA10	.	R	G	.	.	.	.	.	.	.	L	.	.	.	.	.	.	.	.	.	.	.	.	.	.	.	.	.	.	.	.	.	.	.	.	.	.	.	.	.	.	.	.	.	.	.	.	.	.	.	.	.	.	.	.	.	.	.	.	.	.	.	.	.	.	.	.	.	.	.
	8	4663feFE10	.	.	.	.	.	.	.	.	.	.	L	.	.	.	.	.	.	.	.	.	T	.	.	.	.	.	.	.	.	.	.	.	.	.	.	.	.	.	.	.	.	.	.	.	.	.	.	.	.	.	.	.	.	.	.	.	.	.	.	.	.	.	.	.	.	.	.	.	.	.
	2	4663feFH10	.	.	.	.	.	.	.	.	.	.	L	.	.	.	.	.	.	.	.	.	.	.	.	T	.	.	.	.	.	.	.	.	.	.	.	.	.	.	.	.	.	.	.	.	.	.	.	.	.	.	.	.	.	.	.	.	.	.	.	.	.	.	.	.	.	.	.	.	.	.
	1	G4663feFA07	.	.	.	.	.	.	.	.	.	.	L	.	.	.	.	.	G	.	.	.	.	.	.	.	.	.	.	.	.	.	.	.	.	.	.	.	.	.	.	.	.	.	.	.	.	.	.	.	.	.	.	.	.	.	.	.	.	.	.	.	.	.	.	.	.	.	.	.	.	.
	1	4663lib12	.	.	.	.	.	.	.	.	.	.	L	.	.	.	.	.	.	.	.	.	.	.	.	.	.	.	.	.	.	.	.	.	.	.	.	P	.	.	.	.	.	.	.	.	.	.	.	.	.	.	.	.	.	.	.	.	.	.	.	.	.	.	.	.	.	.	.	.	.	.
4664-06 (13)	2	4664inFG06	.	.	.	.	.	.	.	.	.	.	L	.	.	.	.	.	.	.	.	.	.	.	.	.	.	.	.	.	.	.	.	.	.	.	.	.	.	.	.	.	.	.	.	.	V	.	.	.	.	.	.	.	.	.	.	.	.	.	.	.	.	.	.	.	.	.	.	.	.	.
	1	4664inFB06	.	.	.	P	.	.	.	.	.	.	L	.	.	.	.	.	.	.	.	.	.	.	.	.	.	.	.	.	.	.	.	.	.	.	F	.	.	.	.	.	.	.	.	.	.	.	.	.	.	.	.	.	.	.	.	.	T	.	.	.	.	.	.	.	.	.	.	.	.	.
	3	4664afFF06	.	.	.	.	.	.	.	.	.	.	L	.	.	.	.	.	.	.	.	.	.	.	.	.	.	.	.	.	.	.	.	.	.	A	I	.	.	.	.	.	.	.	.	.	.	.	.	.	.	.	.	.	.	I	.	.	.	.	I	.	.	.	.	.	.	.	.	.	.	.
	3	4664afFF11gr6	.	.	.	.	.	.	.	.	.	.	L	.	.	.	.	.	.	.	.	P	.	.	.	.	.	.	.	.	.	.	.	.	.	A	I	.	.	.	.	.	.	.	.	.	.	.	.	.	.	.	.	.	.	I	.	.	.	.	I	.	.	.	.	.	.	.	.	.	.	.
	1	4664afFG06	.	.	.	.	.	.	.	.	.	.	L	.	.	.	.	.	.	.	.	.	.	.	.	.	.	.	.	.	.	.	.	.	.	A	I	.	.	P	.	.	.	.	.	.	.	.	.	.	.	.	.	.	.	I	.	.	.	.	I	.	P	.	.	.	.	.	M	.	.	.
	1	4664afFG11	.	.	.	.	.	.	.	.	.	.	L	.	.	M	.	.	.	.	.	.	.	.	.	.	.	.	.	.	.	.	.	.	.	A	I	.	.	.	.	.	.	.	.	.	.	.	.	.	.	.	.	.	.	I	.	.	.	.	I	.	.	.	.	.	.	.	.	.	.	.
	2	4664afFA11	.	.	.	.	.	.	.	.	.	.	L	.	.	.	.	.	.	.	.	.	.	.	.	.	.	.	.	.	.	.	.	.	.	A	I	.	.	.	.	.	.	.	.	.	.	.	.	.	.	.	A	.	.	I	.	.	.	.	I	.	.	.	.	.	.	.	.	.	.	.
4590-04 (5)	3	4590siFF05	.	.	.	.	.	.	.	.	.	.	L	R	.	.	.	.	.	.	.	P	.	.	.	.	.	.	.	.	M	.	.	.	.	.	.	.	.	.	.	.	.	.	.	.	.	.	.	.	.	.	.	.	.	.	.	.	.	.	.	.	.	.	.	.	.	.	.	.	.	.

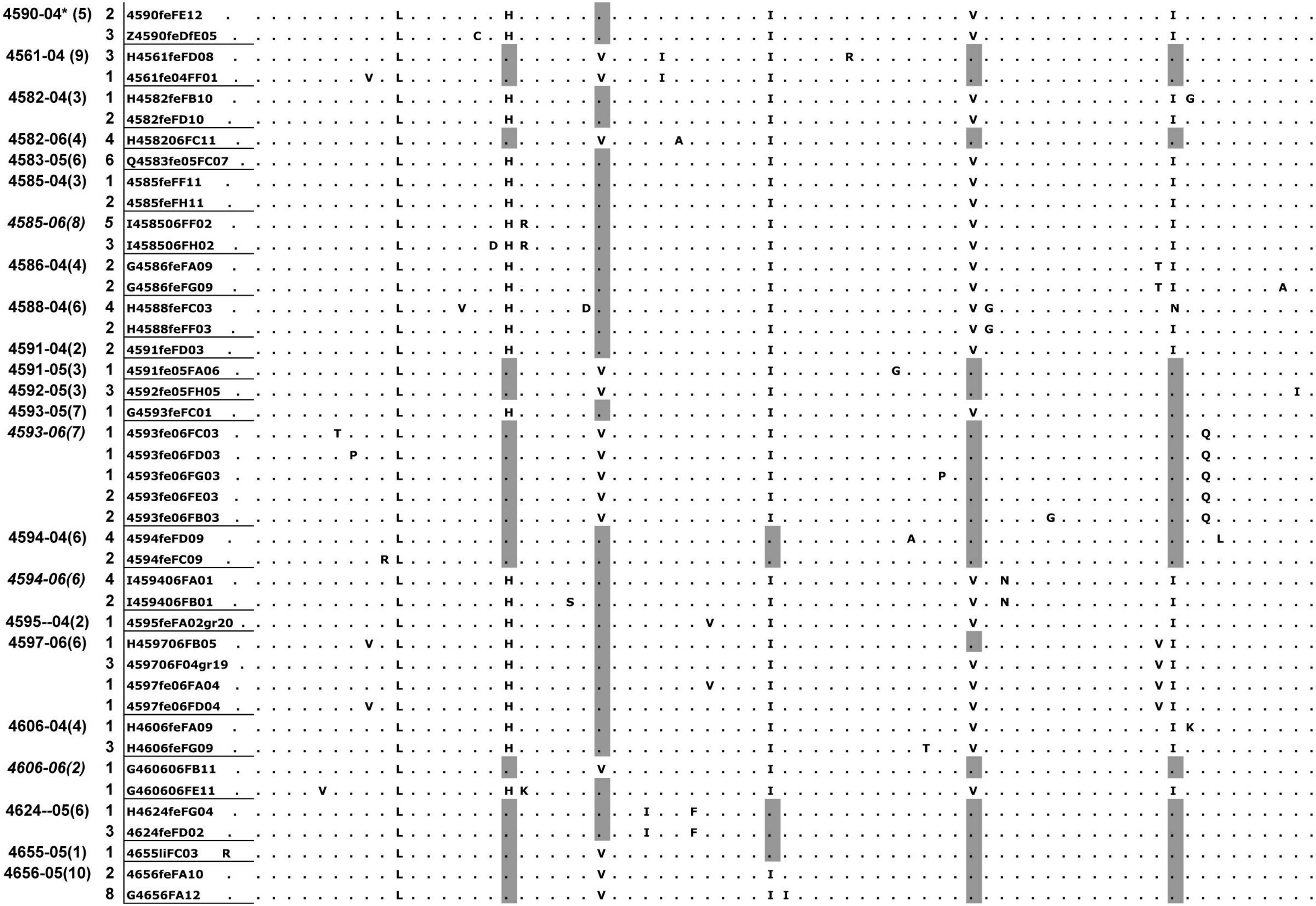

4590-04* (5) 2 4590feFE12
3 Z4590feDfE05
4561-04 (9) 3 H4561feFD08
1 4561fe04FF01
4582-04(3) 1 H4582feFB10
2 4582feFD10
4582-06(4) 4 H458206FC11
4583-05(6) 6 Q4583fe05FC07
4585-04(3) 1 4585feFF11
2 4585feFH11
4585-06(8) 5 I458506FF02
3 I458506FH02
4586-04(4) 2 G4586feFA09
2 G4586feFG09
4588-04(6) 4 H4588feFC03
2 H4588feFF03
4591-04(2) 2 4591feFD03
4591-05(3) 1 4591fe05FA06
4592-05(3) 3 4592fe05FH05
4593-05(7) 1 G4593feFC01
4593-06(7) 1 4593fe06FC03
1 4593fe06FD03
1 4593fe06FG03
2 4593fe06FE03
2 4593fe06FB03
4594-04(6) 4 4594feFD09
2 4594feFC09
4594-06(6) 4 I459406FA01
2 I459406FB01
4595--04(2) 1 4595feFA02gr20
4597-06(6) 1 H459706FB05
3 459706F04gr19
1 4597fe06FA04
1 4597fe06FD04
4606-04(4) 1 H4606feFA09
3 H4606feFG09
4606-06(2) 1 G460606FB11
1 G460606FE11
4624--05(6) 1 H4624feFG04
3 4624feFD02
4655-05(1) 1 4655liFC03
4656-05(10) 2 4656feFA10
8 G4656FA12

Group	n	Sequence	Alignment
4657-05(5)	1	H4657feFC01	.........L..............T.......A............................D........
	1	H4657feFA01	.........L......................A............................D........
	1	4657feFC04	.........L......................A..R.........................D........
	1	H4657feFD01	.........L......................A....................................
	1	H4657feFE01	.........L.....................MA............................D........
4659-05(9)	4	4659feFB11	.........L............V..........I...................................
	1	4659feFE11	.........L............V..........IM..............C...................
	2	4659feFD11	.........L............V..........I.......................I...........
	2	H4659feFD06	.........L............V..........II...............A..................
Aju-92(8)	3	Haju92liFC12	.........L...V........V..
	1	Haju92liFE12	.........L...V...R....V..
	1	Baju92li2FH01	.........L...V........V.....F..
	1	Haju92liFA12	.........L...V........V..............R...............................
	1	Haju92liFB12	.........L...A........V..
	1	Baju92li2FB01	.........L...V........V..
SARS		SARS	NNF..LVFWLW.FV-V.RNTG...CIVMLS..FLR.M.....L-NLTIVTMLVIR.LGCKD.ET..SY
MHV-1		MHV-1	S..IV.I..M.IC.-..ANYL..VTI.SMI...I.G.......L-CMTV..IHTIR.LGFSD..TKHCY
IBV-Beu		IBV-Beu	T.VIL.IV...IGVTPPNGGLV.IT.FASVGI.I.R......SVGITQQCNEMVKN.WLCD..DF.RIR
BCV-K		BCV-K	S..V..I..M.I..-..ANYL..VTI.AMIV..I.G.......L-CM.M..IHTIR.LGYSD..TKHFY
HCV-229E		HCV-229E	K.LFL..V....S.WNWDNFVAFF..S.MV.A.F.RTF.AW.V.-.VQT.YQ.....L.VHN..TV.IS
TGEV		TGEV	...V.....................I...I...I..........-C.....E.....I.MDN...L...
FIPV79-1146		FIPV79-1146	..LV..................V.....................-C.......................

	1	2	3	4	5	7	8	9	11	14	16	18	22	24	26	33	35	36	42	44	45	46	47	48	49	50	55	59	60	61	62	63	64	70	75	77	79	80	86	87	88	89	91	99	101	102	105	113	117	118	120	121	122	130	131	132	134	135	137	140	142	144	150	155	157	158	160	165	166	170
4549coe10	K	A	T	T	V	P	D	L	E	V	T	E	H	V	N	H	I	L	S	V	E	C	E	G	V	E	T	F	Q	N	P	A	H	Y	N	F	S	F	I	V	G	E	N	V	A	V	Q	Y	A	A	L	P	F	R	D	F	H	P	N	H	E	S	V	Y	L	H	Y	K	L	N
4549coe9	.	.	.	.	.	.	.	.	.	.	.	.	.	.	.	.	.	.	.	.	.	.	.	S	.	.	.	.	.	.	.	.	.	.	.	.	.	.	.	.	.	D	.	.	.	.	.	.	.	.	.	.	.	.	.	.	.	.	.	.	.	.	.	.	.	.	.	.	.	.
B4549liDE11	.	.	.	.	.	.	.	.	.	.	A	.	.	.	.	.	.	.	.	.	.	.	.	S	.	.	.	.	.	.	.	.	.	.	.	.	.	.	.	.	.	D	.	.	.	.	.	.	.	.	.	.	.	.	.	.	.	.	.	.	.	.	.	.	.	.	.	.	.	.
4549lic3	.	.	.	.	.	.	.	.	.	.	.	.	.	.	.	.	.	.	.	.	.	.	.	S	.	.	.	.	L	.	.	.	.	.	.	.	.	.	.	.	.	D	.	.	.	.	.	.	.	.	.	.	.	.	.	.	.	.	.	.	.	.	.	.	.	.	.	.	.	.
W4549coDA05	.	.	.	.	.	.	.	.	.	.	.	.	.	.	.	.	.	.	.	.	.	.	.	S	.	.	.	.	.	.	.	.	.	.	.	.	.	.	.	.	.	D	.	.	.	.	.	.	.	.	.	.	.	.	.	.	.	.	.	.	.	.	.	.	.	.	.	.	.	.
B4549liDB11	.	.	.	.	.	.	.	.	.	.	.	.	.	.	.	.	.	.	.	.	.	.	.	S	.	.	.	.	.	.	.	.	.	.	.	.	.	.	.	.	.	D	.	.	.	.	.	.	.	.	.	.	.	.	.	.	.	.	.	.	.	.	.	.	.	.	.	.	.	.
4549lic6	.	.	.	.	.	.	.	.	.	.	.	.	.	.	.	.	.	.	.	.	.	.	.	S	.	.	.	.	.	.	.	.	.	.	.	.	.	.	.	.	.	.	.	.	.	.	.	.	.	.	.	.	.	.	.	.	.	.	.	.	.	.	.	.	.	.	.	.	.	.
A4549liDD02	.	.	.	.	.	.	.	.	.	.	.	.	.	.	.	.	.	.	.	.	.	.	.	S	.	.	.	.	.	.	.	.	.	.	.	.	.	.	.	.	.	V	.	.	.	.	.	.	.	.	.	.	.	.	.	.	.	.	.	.	.	.	.	.	.	.	.	.	.	.
4549lic5	.	.	.	.	.	.	.	.	.	.	.	.	.	.	.	.	.	.	.	.	.	.	.	S	.	.	.	.	.	.	.	.	.	.	.	.	.	.	.	.	.	.	.	.	.	.	.	.	.	.	.	.	.	.	.	.	.	.	.	.	.	.	.	.	.	.	.	.	.	.
4549coe7	.	.	.	.	.	.	.	.	.	.	.	.	.	.	.	.	.	.	.	.	.	.	.	S	.	.	.	.	.	.	.	.	.	.	.	.	.	.	.	.	.	.	.	.	.	.	.	.	.	.	.	.	.	.	.	.	.	.	.	.	.	.	.	.	.	.	.	.	.	.
A4549liDH02	.	.	.	.	.	.	.	.	G	.	.	.	.	.	.	.	.	.	.	.	.	.	.	S	.	.	.	.	.	.	.	.	.	.	.	.	.	.	.	.	.	D	.	.	.	.	.	.	.	.	.	.	.	.	.	L	.	.	.	.	.	.	.	.	.	.	.	.	.	.
W4549coDG05	.	.	.	.	.	.	.	.	.	.	.	.	.	.	.	.	.	.	.	.	.	.	.	S	.	.	.	L	.	.	.	.	.	.	.	.	.	.	.	.	.	.	.	.	.	.	.	.	.	.	.	.	.	.	.	.	.	.	.	.	.	.	.	.	.	.	.	.	.	.
A4549liDC02	.	.	.	.	.	.	.	.	.	.	.	.	.	.	.	.	.	.	.	.	.	.	.	S	.	.	.	.	.	.	.	.	.	.	.	.	.	.	.	.	.	D	.	.	.	.	.	.	.	.	.	.	.	.	.	.	.	.	.	.	.	.	.	.	.	.	.	.	.	.
W4549coDH05	.	.	.	.	.	.	.	.	.	.	.	.	.	.	.	.	.	.	.	.	.	.	.	S	.	.	.	.	.	.	.	.	.	.	.	.	.	.	.	.	.	D	.	.	.	.	.	.	.	.	.	.	.	.	.	.	.	.	.	.	.	.	.	.	.	.	.	.	.	.
B4549liDG11	.	.	.	.	.	.	.	.	.	.	.	.	.	.	.	.	.	.	.	.	.	.	.	S	.	.	.	.	.	.	.	.	.	.	.	.	.	.	.	.	.	D	.	.	.	.	.	.	.	.	.	.	.	.	.	.	.	.	.	.	.	.	.	.	.	.	.	.	.	.
W4549coDB05	.	.	.	.	.	.	.	.	.	.	.	.	.	.	.	.	.	.	.	.	.	.	.	S	.	.	.	.	.	.	.	.	.	.	.	.	.	.	.	.	.	.	.	.	S	.	.	.	.	.	.	.	.	.	.	.	.	.	.	.	.	.	.	.	.	.	.	.	.	.
B4549liDD11	.	.	.	.	.	.	.	.	.	.	.	.	.	.	.	.	.	S	.	.	.	.	.	S	.	.	.	.	.	.	.	.	.	.	.	.	.	.	.	.	.	D	.	.	.	.	.	.	.	.	.	.	.	.	G	.	.	.	.	.	.	.	.	.	.	.	.	.	.	.
4549coe6	.	.	.	.	.	.	.	.	.	.	.	.	.	.	.	.	.	.	.	.	A	.	.	S	.	.	.	.	.	.	.	.	.	.	.	.	.	.	.	.	.	.	.	.	.	.	.	.	.	.	.	.	.	.	.	.	.	.	.	.	.	.	.	.	.	.	.	.	.	.
4549coe8	.	.	.	.	.	.	.	.	.	.	.	.	.	.	.	.	.	.	.	.	.	.	.	S	.	.	.	.	.	.	.	.	.	.	.	.	.	.	.	.	.	D	.	.	.	.	.	.	.	.	.	.	.	.	.	.	.	.	.	.	.	.	.	.	.	.	.	.	.	.
4549lic4	.	.	.	.	.	.	.	.	.	.	.	.	.	.	.	.	.	.	.	A	.	.	.	S	.	.	.	.	.	.	.	.	.	.	.	.	.	.	.	.	.	.	.	.	.	.	.	.	.	.	.	.	.	.	.	.	.	S	.	.	.	.	.	.	.	.	.	.	.	.
4549coe11	.	.	.	.	.	.	.	.	.	.	.	.	.	.	.	.	.	.	.	.	.	.	.	S	.	.	.	.	.	.	.	.	.	.	.	.	.	.	.	.	.	.	.	.	.	.	.	.	.	.	.	.	.	.	.	.	.	.	.	.	.	.	.	.	M	.	.	.	.	.
A4549liDA02	.	.	.	.	.	.	.	.	.	.	.	.	.	.	.	.	.	.	.	.	.	.	.	S	.	.	.	.	.	.	.	.	.	.	.	.	.	.	.	.	.	D	.	.	.	.	.	.	.	.	.	.	.	.	.	L	.	.	.	.	.	.	.	.	.	.	.	.	.	.
A4549liDF02	.	.	.	.	.	.	.	.	.	.	.	.	.	.	.	.	.	.	.	.	.	.	.	S	.	.	.	.	.	.	.	.	.	.	.	.	.	.	.	.	.	.	.	.	.	.	.	.	.	.	.	.	.	.	.	.	.	.	.	.	.	.	.	.	.	.	.	.	.	.
A4549liDB02	E	.	.	.	.	.	.	.	.	.	.	.	.	.	.	.	.	.	.	.	.	.	.	S	.	.	.	.	.	.	.	.	.	.	.	.	.	.	.	.	.	.	.	.	.	.	.	.	.	.	.	.	.	.	.	.	.	.	.	.	.	.	.	.	.	.	.	.	.	.
A4566inDD12	.	.	.	.	.	.	.	.	.	.	.	.	.	.	.	.	.	.	.	.	.	.	.	S	.	.	.	.	.	.	.	.	.	.	.	.	.	.	.	.	.	.	.	.	.	.	.	.	.	.	.	S	.	.	.	.	.	.	.	.	.	.	.	.	.	.	.	.	.	.
A4566inDC12	.	.	.	.	.	L	.	.	.	.	.	.	L	.	.	.	.	.	.	.	.	.	.	S	.	.	.	.	.	.	.	.	.	.	.	.	.	.	.	.	.	.	.	.	.	.	.	.	.	.	.	S	.	.	.	.	.	.	.	.	.	.	.	.	.	.	.	.	.	.
A4566inDE04	.	.	.	.	.	L	.	.	.	.	.	.	.	.	.	.	.	.	.	.	.	.	.	S	.	.	.	.	.	.	.	.	.	.	.	.	.	.	.	.	.	.	.	.	.	.	.	.	.	.	.	S	.	.	.	.	.	.	.	.	.	.	.	.	.	.	.	.	.	.
4618lnDG12	.	.	.	.	.	.	.	.	.	.	.	.	.	L	.	.	.	.	.	.	.	.	.	S	.	.	.	.	.	.	S	.	.	.	.	.	.	.	.	.	.	.	.	.	.	.	.	.	.	.	.	.	.	.	.	.	.	.	.	.	.	.	.	.	.	.	.	.	.	.
4618lig8	.	.	.	.	.	.	.	.	.	.	.	.	.	L	.	.	.	.	.	.	.	.	.	S	.	.	.	.	.	.	S	.	.	.	.	.	.	.	.	.	.	.	.	.	.	.	.	.	.	.	.	.	.	.	.	.	.	.	.	.	.	.	.	.	.	.	.	.	.	.
4618lig12	.	.	.	.	.	.	.	.	.	.	.	.	.	L	.	.	.	.	.	.	.	.	.	S	.	.	.	.	.	.	S	.	.	.	.	.	.	.	.	.	.	.	.	.	.	.	.	.	.	.	.	.	.	.	.	.	R	.	.	.	.	.	.	.	.	.	.	.	.	.
4618lnDE12	.	.	.	.	.	.	.	.	.	.	.	.	.	L	.	.	.	.	.	.	.	.	.	S	.	.	.	.	.	.	S	.	.	.	.	.	.	.	.	.	.	.	.	A	.	.	.	.	.	.	.	.	S	.	.	.	.	.	.	.	.	.	.	.	.	.	.	.	.	.
4618lig4	.	.	.	.	.	.	.	.	.	.	.	.	.	L	.	.	.	.	.	.	.	.	.	S	.	.	.	.	.	.	S	.	.	.	.	.	.	.	.	.	.	.	.	.	.	.	.	.	.	.	.	.	.	.	.	.	.	.	.	.	.	.	.	.	.	.	.	.	.	.
4618lnDC12	.	.	.	.	.	.	.	.	.	.	.	.	.	L	.	.	.	.	.	.	.	.	.	S	.	.	.	.	.	.	S	.	.	.	.	.	.	.	.	.	.	.	.	.	.	.	.	.	.	.	.	.	.	.	.	.	.	.	.	.	.	.	.	.	.	.	.	.	.	.
4618lig6	.	.	.	.	.	.	.	.	.	.	.	.	.	L	.	.	.	.	.	.	.	.	.	S	.	.	.	.	.	.	S	.	.	.	.	.	.	.	.	.	.	.	.	.	.	.	.	.	.	.	.	.	.	.	.	.	.	.	.	.	.	.	.	.	.	Y	.	.	.	.
4618liDE04c	.	.	.	D	.	H	.	.	.	.	.	.	.	.	H	.	.	.	.	.	.	.	.	S	.	.	.	.	.	.	.	.	.	.	.	L	.	.	.	.	.	.	.	.	.	.	.	H	.	V	.	.	.	.	.	.	Y	.	.	.	.	T	.	.	.	.	.	.	.	D
4618lnDD12	.	.	.	.	.	.	.	.	.	.	.	.	.	L	.	.	.	.	.	.	.	.	.	S	.	.	.	.	.	.	S	.	.	.	.	.	.	.	.	.	.	.	.	.	.	.	.	.	.	.	.	.	.	.	.	.	.	.	.	.	.	.	.	.	.	.	.	.	.	.
4618lig5	.	.	.	.	.	.	.	.	.	.	.	.	.	L	.	.	.	.	.	.	.	.	.	S	.	.	.	.	.	.	S	.	.	.	.	.	.	.	.	.	.	.	.	.	.	.	.	.	.	.	.	.	.	.	.	.	.	.	.	.	.	.	.	.	.	.	.	.	.	.
4618lnDF12	.	.	.	.	.	.	.	.	.	.	.	.	.	L	.	.	.	.	.	.	.	.	.	S	.	.	.	.	.	.	S	.	.	.	.	.	.	.	.	.	.	.	.	.	.	.	.	.	.	.	.	.	.	.	.	.	.	.	.	.	.	.	.	.	.	.	.	.	.	.
4653spDE11	.	.	.	A	.	N	.	P	T	T	.	.	N	.	H	.	V	.	.	.	.	.	.	S	I	D	.	.	.	K	S	.	.	F	.	.	.	.	.	.	.	.	.	.	.	.	E	H	.	V	.	.	.	.	T	.	.	A	.	Y	.	N	.	.	.	.	.	.	.	D
4653lid5	.	.	.	A	.	N	.	.	T	T	.	.	N	.	H	.	V	.	.	.	.	.	.	S	I	D	.	.	.	K	S	.	.	F	.	.	.	.	.	.	.	.	.	.	.	.	E	H	.	V	.	.	.	.	T	.	.	A	.	Y	.	N	.	.	.	.	.	.	.	D
B4653spDC12	.	.	.	.	.	.	.	.	.	.	.	.	.	L	.	.	.	.	.	.	.	.	.	S	.	.	.	.	.	.	S	.	.	.	.	.	.	.	.	.	.	.	.	.	.	.	.	.	.	.	.	.	.	.	.	.	.	.	.	.	.	.	.	.	.	.	.	.	.	.
4653lid3	.	.	.	A	.	N	.	.	T	T	.	.	N	.	H	.	V	.	.	.	.	.	.	S	I	D	.	.	.	K	S	.	.	F	.	.	.	.	.	.	.	.	.	.	.	.	G	H	.	V	.	.	.	.	T	.	.	A	.	Y	.	N	.	.	.	.	.	.	.	D
4653lid2	.	.	.	A	.	N	.	.	T	T	.	.	N	.	H	.	V	.	.	.	.	.	.	S	I	D	.	.	.	K	S	.	.	F	.	.	.	.	.	.	.	G	.	.	.	.	E	H	.	V	.	.	.	.	T	.	.	A	.	Y	.	N	.	.	.	.	.	.	.	D
B4653spDG12	.	.	.	A	M	N	.	.	T	T	.	.	N	.	H	.	V	.	.	.	.	.	.	S	I	D	.	.	.	K	S	.	.	F	.	.	.	.	.	.	.	.	.	.	.	.	E	H	.	V	.	.	.	.	T	.	.	A	.	Y	.	N	.	.	.	.	.	.	.	D
4653spDD11	.	.	.	A	.	N	.	.	T	T	.	.	N	.	H	.	V	.	.	.	.	.	.	S	I	D	.	.	.	K	S	.	.	F	.	.	.	.	.	.	.	.	.	.	.	.	E	H	.	V	.	.	.	.	T	.	.	A	.	Y	.	N	.	.	.	.	.	.	.	D
4653spDH11	.	.	.	A	.	N	.	.	T	T	.	.	N	.	H	.	V	.	.	.	.	.	.	S	I	D	.	.	.	K	S	.	.	F	.	.	.	.	.	.	.	.	.	.	.	.	E	H	.	V	.	.	.	.	T	.	.	A	.	Y	.	N	.	.	.	.	.	.	.	D
4653lid4	.	.	.	A	.	N	.	.	T	T	.	.	N	.	H	.	V	.	.	.	.	.	.	S	I	D	.	.	.	K	S	.	.	F	.	.	.	.	.	.	.	.	.	.	.	.	E	H	.	V	.	.	.	.	T	.	.	A	.	Y	.	N	.	.	.	.	.	.	.	D

```
4653spDB11    ...A.N..TT..N.H.V......SID...KS..F............EH.V....T..A.Y.N.......D
4653spDG11    ...A.N..TT..N.H.V......SID..RKS..F............EH.V....T..A.Y.N.......D
4653lid12     ...A.N..TT..N.H.V......SID...KS..F............EH.V....T..A.Y.N.......D
4653lid9      ...A.N..TT..N.H.V......SID...KS..F............EH.V....TL.A.Y.N.......D
4662jeDG10    ...D.H........H........S.......................H.V...........T.......D
4662lif2      ...D.H........H........S.......................H.V...........T.......D
4662lif3      ...D.H........H........S.......................H.V...........T.......D
4662jeDD10    ...D.H........HR.......S.......................H.V...........T.......D
4662jeDH10    ...D.H........H........S.......................H.V...........T.......D
4662jeDC10    ...D.H........HR.....R.S.......................H.V...........T.......D
A4663spDE03   .....NN.......H.VS.....S.........H....VI.......H.V......R...DT........
E4663feDD05   ...D.H........H........S.......................H.V...........T.......D
4663lia5      ...D.H........H........S.......................H.V...........T.......D
E4663feDE05   ...D.H........H........S.......................H.V...........T.......D
A4663spDF03   .....NN.......H.V......S.........H....VI.......H.V..........DT.H......
A4663liDC05   ...D.H........H........S.......................H.V......Y....T.......D
A4663liDD05   ...D.H........H........S.......................H.V......Y....T.......D
4663lia11     ...D.H........H........S.......................H.V...........T.......D
A4663spDH03   .....NN.......H.V......S.........H....VI.......H.V......R...DT........
A4663liDG05   ...D.H........H......R.S.......................H.V...........T.......D
E4663feDG05   ...D.H........H........S.......................H.V..L........T.......D
4663lia4      ...D.H........H........S.......................H.V...........T.......D
A4663spDA03   .....NN.......H.V......S.........H....VI.......H.V..........DT........
A4663liDB05   ...D.H........H........S.......................H.VP.....Y....T.......D
A4663liDF05   ...D.H........H........S..............L........H.V...........T.......D
A4663spDC03   .....NN.......H.V......S.........H...LVI.......H.V......R...DT........
4663lia9      ...D.H........H........S.......................H.V...........T.......D
4664inDB02    ...D.H........H........S.......................H.V...........T.......D
4664inDD02    ...D.H........H........S..A....................H.V...........T.......D
4664inDC02    ...D.H........H........S.......................H.V...........T......PD
4590siDC08    .............L..V......S......S.......................................
4590siDD08    .............L.........S......S.......................................
4590siDB08    .............L.........S......S.......................................
4590siDE08    .............L.........S......S.......................................
4590siDG09    .............L.........S......S.......................................
4590siDA07    .............L.........S......S............A.A........................
4590siDF08    .............LD........S......S.......................................
4590siDD09    .............L.........S......S.......................................
4590siDC07    .............L.........S......S.......................................
4590siDE07    .............L.........S......S....................................R..
4590siDB07    .............L.........S......S.......................................
4590siDA08    .............L.........S......S.......................................
```

```
D4590feDH05   .....NN.......H.V......S.........H....VI.......H.V..........DT........
B4590feDD04   .....NN.......H.V......S.........H....VI.......H............DT...P....
D4590feDE07   .....NN.......H.V......S.........H....VI.......H.V..........DT........
D4590feDC06   ....ANN.......H.V......S..A......H....VI.......H.V..........DT........
D4590feDF09   .....NN.......H.V......S.........H....VI.......H.V.....L....DT........
B4590feDB04   .....NN.......H.V......S.........H....VI.......H.V..........DT........
D4590feDB09   .....NN.......H.V.....GS.........H....VI.......H.V..........DT........
D4590feDG07   .....NN.......H.V......S.........H....VI.......H.VP.........DT........
```

```
D4590feDC05    . . . . . N N . . . . . . . H . V . . . . . . S . . . . . . . . . H . . . . V I . . . . . . . H . V . . . . . . . . . . D T . . . . . . . .
D4590feDF05    . . . . . N N . . A . . . . H . V . . . . . . S . . . . . . . . . H . . . . V I . . . . . . . H . V . . . . . . . . . . D T . . . . . . . .
D4590feDG05    . . . . . N N . . . . . . . H . V . . . . . . S . . . . . . . . . H . . . . V I . . . . . . . H . V . . . . . . . . . . D T . . . . . . . .
D4590feDA08    . . . . . N N . G . . . . . H . V . . . . . . S . . . . . . . . . H . . . . V I . . . . . . . H . V . . . . . . . . . . D T . . . . . . . .
B4590feDE04    . . . . . N N . . . . . . . H . V . . . . . . S . . . . . . . . . H . . . . V I . . . . . . . H . V . . . . . . . . . . D T . . . . . . . .
D4590feDC03    . . . . . N N . . . . . . . H . V . . . . . . S . . . . . . . . . H . . T . V I . . . . . . . H . V . . . . . . . . . . D T . . . . . . . .
D4590feDD09    . . . . . N N . . . . . . . H . V . . . . . . S . . . . . . . . . H . . . . V I . . . . . . . H . V . . . . . . . . . . D T . . . . . . . .
D4590feDG06    . . . . . N N . . . . . . . H . V . . . . . G S . . . . . . . . . H . . . . V I . . . . . . . H . V . . . . . . . . . . D T . . . . . . . .
D4590feDF08    . . . . . N N . . . . . . . H . V . . . . . . S . . . . . . . . . H . . . . V I . . . . . . . H . V . . L . . . . . . . D T . . . . . . . .
D4590feDF04    . . . . . N N . . . . . . . H . V . . . . . . S . . . . . . . . . H . . . . V I . . . . V . . H . V . . . . . . . . . . D T . . . . . . . .
D4590feDE03    . . . . . N N . . . . . . . H . V . . . . . . S . . . . . . . . . H . . . . V I . . . . . . . H . V . . . G . . . . . . D T . . . . . . . .
D4590feDC07    . . . . . N N . . . . . . . H . V . . . . . . S . . . . . . . . . H . . . . . I . . . . . . . H . V . . . . . . . . . . D T . . . . . . . .
B4590feDA04    . . . . . N N . . . . . . . H . V . . . . . . S . . . . . . . . . H . . . . V I . . . . . . . H . V . . . . . . . .   . D T . . . . . . . .
S4581feDE10    . . . . . N N . . . . . . . H . V . . . . . . S . . . . . . . . . H . . . . V I . . . . . . . H . V . . . . . . . . S . D T . . . . . . . .
S4581feDH10    . . . . . N N . . . . . . . H . V . . . . . . S . . . . . . . . . H . . . . V I . . . . . . . H . V . . . . . L . . S . D T . . . . . . . .
S4581feDC10    . . . . . N N . . . . . . . H . V . . . . . . S . . . . . . . . . H . . . . V I . . . . . . . H . V . . . . . . . . S . D T . . . . . . . .
E458306DB04    . . . . . N N . . . . . . . H . V . . . . . . S . . . . . . . . . H . . . . V I . . . . . . . H . V . . . . . . . . S . D T . . . . . . . .
E458306DE04    . . . . . N N . . . . . . . H . V . . . . . . S . . . . . . . . . H . . . . V I . . . . . . . H V V . . . . . . . . S . D T . . . . . . . .
E458306DG04    . . . . . N N . . . . . . . H . V . . . . . . S . . . . . . . . . H . . . . V I . . . . . . . H . V . . . . . . . . S . D T . . . . . . . .
E458306DD04    . . . . . N N . . . . . . . H . V . . . . . . S . . . . . . . . . H . . . . V I . K . . . . . H V V . . . . . . . . S . D T . . . . . . . .
E458306DC04    . . . . . N N . . . . . . . H . V . . . . . . S . . . . . . . . . H . . . . V I . . . . . . . H V V . . . . . . . . S . D T . . . . . . . .
S4584feDD09    . . . . . N N . . . . . . . H . V . . . . . . S . . . . . . . . . H . . . . V I . . . . V . . H . V . . . . . . . . S . D T . . . . . . . .
S4584feDB09    . . . . . N N . . . . . . . H . V . . . . . . S . . . . . . . . . H . . . . V I . . . . V . . H . V . . . . . . . . S . D T . . . . . . . .
S4584feDG09    . . . . . N N . . . . . . . H . V . . . . . . S . . . . . . . . . H . . . . V I . . . . V . . H . V . . . . . . . . S . D T . . . . . . . .
E4586feDF07    . . . . . N N . . . . . . . H . V . . . . . . S . . . . . . . . . H . . . . V I . . . . . . . H . V . . . . . . . . S . D T . . . . . . . .
E4586feDE07    . . . . . N N . . . . . . . H . V . . . . . . S . . . . . . . . . H . . . . V I . . . . . . . H . V . . . . . . . . S . D T . . . . . . . .
E4586feDD07    . . . . . N N . . . . . . . H . V . . . . . . S . . . . . . . . . H . . . . V I . . . . . . . H . V . . . . . . . . S . D T . . . . . . . .
E4589feDF06    . . . . . N N . . . . . . . H . V . A . . . . S . . . . . . . . . H . . . . V I . . . . . . . H . V . . . . . . . .   . D T . . . . . . . .
E4591feDF12    . . . . . S N . . . . . . . H . V . . . . . . S . . . . . . . . . H . . . . V I . . . . . . . H . V . . . . . . . . S . D T . . . . . . . .
E4591feDG12    . . . . M N N . . . . . . . H . V . . . . . . S . . . . . . . . . H . . . . V I . . . . . . . H . V . . . . . . . . S . D T . . . . . . . .
S4591feDD06    . . . . . N N . . . . . . . H . V . . . . . . S . . . . . . . . . H . . . . V I . . . . . . . H . V . . . . . . . . S . D T . . . . . . . .
4591feh11      . . . . . N N . . . . . . . H . V . . . . . . S . . . . . . . . . H . . . . D I . . . . . . . H . V . . . . . . . . S . D T . . . . . . . .
4591feh7       . . . . . N N . . . . . . . H . V                                 H . . . . V I . . . . . . . H . V . . . . . . . . S . D T . . . . . . . .
W4591feDH06    . . . . . N N . . . . . . . H . V . . . . . . S . . . . . . . . . H . . . . V I . . . . . . . H . V . . . . . . . . S . D T . . . . . . . .
W4591feDF06    . . . . . N N . . . S . . . H . V . . . . . . S . . . . . . . . . H . . . . V I . . . . . . . H . V . . . . . . . . S . D T A . . . . . . .
E4591feDB12    . . . . . N N . . . . . . . H . V . . . . . . S . . . . . . . . . H . . . . V I . . . . . . . H . V . . . . . . . . S . D T . . . . . . . .
4591feh12      . . . . . N N . . . . . . . H . V . . . . . . S . . . . . . . . . H . . . . V I . . . . . . . H . V . . . . . . . . S . D T A . . . . . . .
E4591feDE12    . . . . . N N . . . . . . . H . V . . . . . . S . . . . . . . . . H . . . . V I . . . . . . . H . V . . . . . . . . S . D T . . . . . . . .
W4591feDB06    . . . . . N N . . . . . . . H . V . . . . . . S . . . . R . . . . H . . . . V I . . . . . . . H . V . . . . . . . . S . D T . . . . . . . .
4591feh10      . . . . . N N . . . . . . . H . V . P . . . . S . . . . . . . . . H . . . . V I . . . . . . . H . V . . . . . . . . S . D T . . . . . . . .
E4591feDC12    . . I . . N N . . . . . . . H . V . . . . . . S . . . . . . . . . H . . . . V I . . . . . . . H . V . . . . . . . . S . D T . . . . . . . .
S4591feDG06    . . . . . N N . . . . . . . H . V . . . . . . S . . . . . . . . . H . . . . V I . . . . . . . H . V . . . . . . . . S . D T . . . . . . . .
S4594fe06DD    . . . . . N N . . . . . . . H . V . . . . . . S . . . . . . . . . H . . . . V I . . . . . . . H . V . . . . . . . . S . D T . . . . . . P .
S4594fe06DF    . . . . . N N . . . . . . . H . V . . I . . . S . . . . . . . . . H . . . . V I . . . . . . . H . V . . . . . . . . S . D T . . . . . . . .
S4594fe06DB    . . . . . N N . . . . . . . H . V . . . . . . S . . . . . . . . . H . . . . V I . . . . . . . H . V . . . . . . . . S . D T . . . . . . . .
S4594fe06DE    . . . . . N N . . . . . . . H . V . . . . . . S . . . . . . . . . H . . . . V I . . S . . . . H . V . . . . . . . . S . D T . . . . . . . .
S4594feDC12    . . . . . N N . . . . . . . H . V . . . . . . S . . . . . . . . . H . . . . V I . . . . . . . H . V . . . . . . . . . . . . . . . . . . . .
S4595feDF11    . . . . . N N . . . . . . . H . V . . . . . . S . . . . . . . . . H . . . . V T . . . . . . . H . V . . . . . . . . S . D T . . . N . . . .
E4595feDE01    . . . . . N N . . . . . . . H . V . . . . . . S . . . . . . . . . H . . . . V I . . . . . . . H . V . . . . . . . . S . D T . . . N . . . .
E4595feDF01    . . . . . N N . . . . . . . H . V . . . . . . S . . . . . . . . . H . . . . V I . . . . . A . H . V . . . . . . . . S . D T . . . . . . . .
```

S4595feDD11	. . . . . N N . . . . H . V . . . . . . S . . . . H . . . . V I . . . . H . V . . . . S . D T . . . .
S4595feDE11	. . . . . N N . . . . H . V . . . . . . S . . . . Q H . . . . V I . . . . H . V . . . . S . D T . . . .
S4595feDH11	. . . . . N N . . . . H . V . . . . . . S . . . . H . . . . V I . . . . H . V . . . . S . D T . . . N . . . .
S4595feDG11	. . . . . N N . . . . H . V . . . . . . S . . . . H . . . . V T . . . . H . V . . . . S . D T . . . .
S4597fe06DC	. . . . . N . . . . H . V . . . . . . S . . . . L . L . . V I . . D . . . . H . V . . . .
S4597fe06DG	. . . . . I . . . . H . V . . . . . . S . . . . F . . . . V I . . . . H . V . . . .
S4597fe06DA	. . . . . N . . . . H . V . . . . . . S . . . . F . . . . V I . . . . H . V . . . .
S4597fe06DF	. . . . . N . . . . H . V . . . . . . S . . . . L . . . . V I . . D . . . . H . V . . . .
S4597fe06DD	. . . . . N . . . . H . V . . . . . . S . . . . L . . . . V I . . D . . . . H . V . . . .
S4597fe06DE	. . . . . N . . . . H . V . . . . . . S . . . . L . . . . V I . . D . . . . H . V . . . .
E460606DF09	. . . . . N N . . . . H . V . . . . . . S . . . . H . . . . V I . . . . H . V . . . . S . D T . H . . . . . .
E460606DC09	. . . . . N N . . . . H . V . . . . . . S . . . . H . . . . V I . . . . H . V . . . . S . D T . H . . . . . .
E460606DE09	. . . . . N N . . . . H . V . . . . . . S . . . . H . . . . V I . . D . . . . H . V . . . . S . D T . H . . . . . .
E4609feDC02	. . . . . H . . . . S . . . .
E4609feDB02	. . . . S . . . .
E4609feDE02	. . . . S . . . . V . . . .
E4609feDH02	. . . . S . . . .
E4609feDA02	. . . . S . . . . V . . . . C . . . . . .
E4612feDA08	. . . . . N N . . . . H . V . . . . . . S . . . . H . . . . V I . . . . H . V . . . . S . D T . . . . . R . .
E4612feDB08	. . . . . N N . . . . H . V . . . . . . S . . . . H . . . . V I . . . . H . V . . . . S . D T . . . .
E4612feDG08	. . . . . N N . . . . H . V . . . . . . S . . . . H . . . . V I . . . . H . . . . L . . . . . S . D T . . . . . . Q .
E4612feDD08	. . . . . N N . . . . H . V . . . . . . S . . . . H . . T . V I . . . . H . . . . S . D T . . . . . T . .
E4612feDC08	. . . . . N N . . . . H . V . . . . . . S . . . . H . . . . V I . . . . H . V . . . . S . D T . . . .
E4612feDF08	. . . . . N N . . . . H . V . . . . . . S . . . . H . . . . V I . . . . H . V . . . . S . D T . . . .
S4624feDB05	. . I D . H . P . . . D . I H . . . . S . . . . . D . . . . S . . . . . . H . V . . . Q Y . Y . . . . A . H . . . . . .
S4624feDF05	. . I D . H . P . . . D . I H . . . . S . . . . . D . . . . S . . . . . . H . V . . . Q Y . Y . . . . A . H . . . . . .
S4624feDH05	. . I D . H . P . . . D . I H . . . . S . . . . . D . . . . S . . . . A . H . V . . . Q Y . Y . . . . A . H . . . . . .
S4656feDD07	. . N . . Y . . . . H . . . . D S . . . . H . . . . G . . . . . H . V . . . . T . H . . . . . .
E4656feDC10	. . N . . Y . . . . H . . . . D S . . . . H . . . . G . . . . . H . V . . . . T . H . . . . . .
E4656feDA10	. . N . . Y . . . . H . . . . D S . . . . H . . . . G . . . . . H . V . . . . T . H . . . . . .
S4656feDA07	. . N . . Y . . . . H . . . . D S . . . . . D . . . H . . . . G . . . . . H . V . . . . T . H . . . . . .
E4657feDA11	. . . D . Y . . . . I H . V . F I V . . S I . . L . E . . Y . . . . H . V . . . . S Y . T . . M . H . . D
E4657feDE11	. . . D . Y . . . . I H . V . F I V . . S I . . L . E S . Y . . . . H . V . . . . S Y . T . . M . H . . D
E4657feDB11	. . . D . Y . . . . I H . V . F I V . . S I . . L . E S . Y . . . . H . V . . . . S . . T . . M . H . . D
S4659feDG08	. . N . . Y . . . . H . . . . D S . . . . H . . . . . . D G . . . . . H . V . . . . T . H . . . . . .
FECVUCD	R . . A A D . . . . H . . . . S . . . . . . F . . H . . . . . . S . . . . . . H . V . . . Q . . . . S . . T . H M . . . . D
FIPVUCD1	. V . A A N . . . . H . . . . S . . . . H . . . . . . S . . . . . . H . V . . . P . . Y . S . . T . H M . . . . D
FIPV791146	. . . A . N . . . . . D . I H . V . . . . I . . . . D . . . . S . . . . . . H . V . . . . D . D T . H . . . R . D
FIPVUCD3	R . . A A D . . . . H . . . . S . . . . . . F . . H D . . . . . S . . . . . . H . V . . . Q . . . . S . . T . H M . . . . D
Aaju92liDH1	1 T . A . Y . . . . N . Y . H S . . . I . . D S I . . . . S . . . . S . . . . I . . . . . M . . . V . . . . D T . H . N . . . D
Aaju92liDC1	1 T . A . Y . . . . N . Y . H S . . . I . . D S I . . . . S . . . . S . . . . I . Q . . . M . . . V . . . . D T . H . N . . . D
Aaju92liDD1	1 T . A . Y . . . . N . Y . H S . . . I . . D S I . . . . S . . . . S . . . . I . . . . . M . . . V . . . . D T . H . N . . . D
Aaju92liDF1	1 T . A . Y . . . . N . Y . H S . . . I . . D S I . . . . S . . . . S . . . . I . G . . . M . . . V . . . . D T . H . N . . . D
