# Supplementary figures and images for: Genetics and Pathogenesis of Feline Infectious Peritonitis Virus
Source: Emerg Infect Dis. 2009 Sep;15(9):1445–52. doi: 10.3201/eid1509.081573 (PMC2819880; doi:10.3201/eid1509.081573)

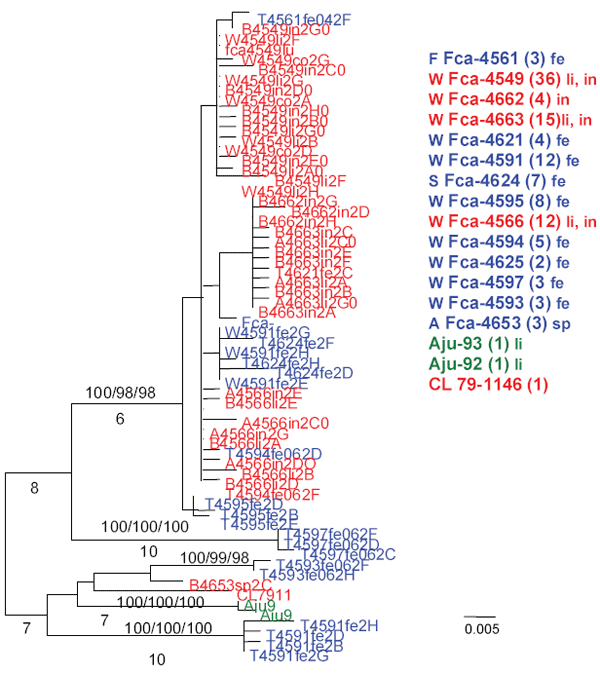

Supplement: Appendix Figure 2 — Midpoint rooted maximum likelihood (ML) tree of unique pol1a 386-bp sequences (ML -ln L = 1300.12586 best tree found by maximum parsimony: length = 125, confidence interval = 0.832, retention index = 0.926). Cloned sequences from feline infectious peritonitis (FIP) cases are shown in red, feline enteric coronavirus (FECV) asymptomatic cats are shown in blue, and feline coronavirus (FCoV) virulent strain from Aju-92 (cheetah) is in green. Each sequence is labeled as follows: source farm (W, Weller Farm; F, Frederick Animal Shelter; S, Seymour Farm; M, Mount Airy Shelter; A, Ambrose Farm), 4-digit cat identification number, tissue source (fe, feces; af, ascites fluid; co, colon; li, liver; sp, spleen; in, intestine; je, jejunum; ln, lymph node), 2-digit year (e.g., 04 = 2004), and number of clones for each sequence. Scale bar indicates number of substitutions per site. [file 08-1573_appF2-s5.gif]

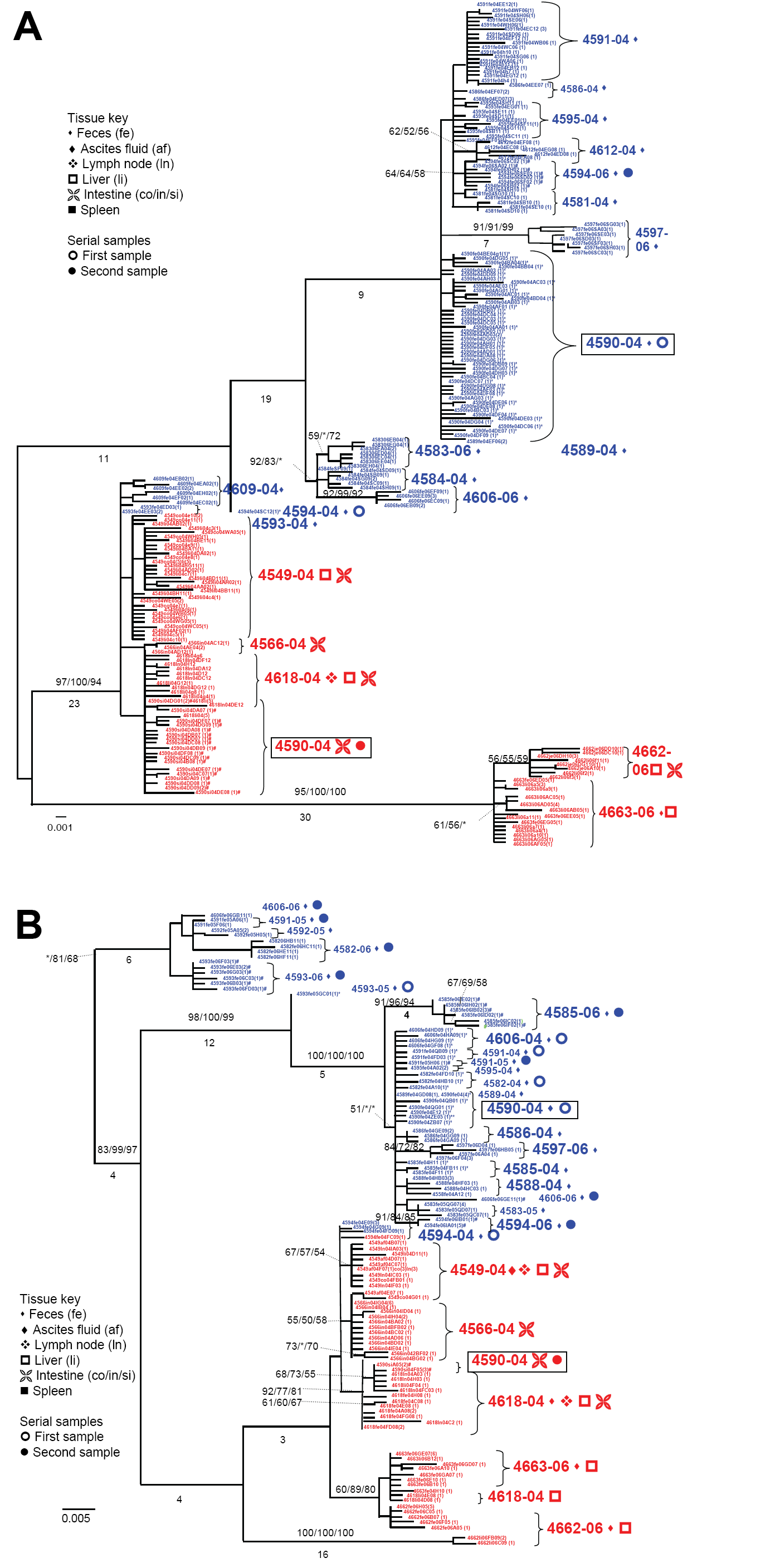

Supplement: Appendix Figure 3 — Feline coronavirus (FCoV) sequence from cats from the Weller farm. Maximum likelihood phylogenetic tree of unique membrane and nonstructural protein 7b (NSP 7b) FCoV gene sequences showing monophyly correlating to disease status. Cloned sequences from feline infectious peritonitis virus (FIPV) biotypes are shown in red; feline enteric coronavirus (FECV) biotypes are shown in green. A) membrane (maximum likelihood [ML] -ln L = 2646.84352 best tree found by maximum parsimony (MP): length = 270, CI = 0.789, retention index [RI] = 0.971; mid-point rooted); B) NSP 7b (ML -n L= 3997.98885 best tree found by MP: length = 411, confidence interval [CI] = 0.791, RI = 0.981; mid-point rooted). The number of cats is indicated in parenthesis in the key. Each sequence is labeled as follows: 4-digit cat identification number, tissue source (fe, feces; af, ascites fluid; co, colon; li, liver; sp, spleen; in, intestine; je, jejunum; ln, lymph node), 2-digit year (e.g., 04 = 2004), and the unique 3-4-digit sequence number. The number of clones for each sequence is indicated after the sequence label in parenthesis. Where ML tree was congruent with MP tree, branch lengths are indicated below branches; the number of homoplasies is in parenthesis after the branch length. Bootstrap values are shown (MP/minimum evolution/ML) above branches. Virus sequence obtained from cat 4590 in May 2004 and at the time of death due to FIPV in December 2004 is indicated by box. The 2 distinct virus genotypes isolated from this case pre- and postdisease in both the membrane and NSP 7b genes are consistent with the dual circulating virulent and avirulent strains in FCoV pathogenesis. Scale bars indicate substitutions/site. [file 08-1573_appF3-s6.gif]
